# Empirical Evidence for the Continuing Need to ‘Think Small First’ in UK Company Law

**DOI:** 10.1007/s40804-022-00258-y

**Published:** 2022-08-11

**Authors:** Jonathan Hardman, Guillem Ramírez Santos

**Affiliations:** 1 School of Law, University of Edinburgh, Edinburgh, UK; 2 School of Informatics, University of Edinburgh, Edinburgh, UK

**Keywords:** Company law, Corporate law, Empirical methodology, Think small first

## Abstract

The most recent UK corporate law restatement has its stated aim to ‘think small first’ in company law legislation. This article is the first to use data science and imaging techniques to provide an empirical snapshot of the entire UK corporate database. It identifies the continuing need to think small first: most companies are small when tested by corporate type (public v private) and type of accounts publicly filed. We then factor in time series, which evidences that most companies are newer and smaller companies. This article then identifies the implications of this novel empirical analysis. First, corporate law analysis tends to ‘think big first’, and will either need to justify such an approach or change it. Second, a large number of companies provide no public financial information due to inherent time lag. The sheer scale of new companies challenges this approach. Third, the UK should provide a corporate governance framework for smaller companies. Fourth, the UK’s corporate accounting regime thinks small first in substance, but its form needs to be simplified to truly think small first. Fifth, whilst more mortgages were granted by smaller companies, larger companies granted more mortgages per company: so arguably corporate finance bucks the trend for the need to think small first.

## Introduction

When the UK’s corporate law regime was last restated in 2006,^1^ one of the UK government’s overarching aims was to ‘think small first’.^2^ Thinking small first is a mindset that encourages corporate law legislation to be focused on the smallest companies first, with added complexity included as companies get larger. It has been said that this arose due to the vast majority of UK companies prior to 2000 being small private companies, ‘so that from the economic perspective the role of such companies is critical in laying the foundations for future growth’.^3^ However, it is generally held that legal developments in UK company law had historically been targeted at larger companies rather than smaller companies.^4^ The UK government identified requirements as to the filing of accounts, in particular, as being too complex for private companies.^5^ A number of reforms were made, including requiring fewer meetings for private companies and making arranging meetings easier,^6^ providing a more straightforward constitution for private companies,^7^ and making it easier for private companies to return capital to their shareholders.^8^ Reports and accounting were meant to be simplified for smaller companies,^9^ and the public register was reformed to make it easier for smaller companies to operate.^10^ The ‘think small first’ mantra for reform has been adopted at European level,^11^ although it has been argued that it has not been consistently deployed.^12^ Simplification of corporate registration regimes to encourage small businesses to incorporate has been followed globally.^13^

The purpose of this article is to use empirical analysis to argue that there is a prima facie case that the UK should continue to ‘think small first’ in respect of company law reform, and it argues that legislatively and academically it fails to do so.^14^ Empirical analysis lets us ‘test our basic assumptions about the world’.^15^ This article uses empirical techniques from data science to review the entire UK corporate register as at 1 June 2021 (downloaded on 3 June 2021) and explore the continued need to think small first within the UK. This holistic empirical approach is novel in the context of UK corporate law. A snapshot of the UK’s corporate register is released once a month. It provides both the numerical overview of how many companies have been added to and removed from the register,^16^ and certain data in respect of each company on the corporate register.^17^ The latter contains nearly 5,000,000 records and is therefore available in 6 spreadsheets, or together as one file in CSV format. We used the fuller data provided in this snapshot of the UK registry as at 1 June 2021. The data listed includes whether the company is a private or public company (or any other company type),^18^ the company’s name,^19^ the company’s registered number,^20^ its registered office address,^21^ account type,^22^ and the number of mortgages that have been granted by the company.^23^ Given the sheer number of records that are included, the challenge here lies in computational scale rather than complexity of computational methods.^24^ This is the first article to provide such a holistic quantitative analysis of the entire UK corporate database as at a specific time, and to identify its implications for UK corporate law and more broadly. By including all UK companies, it avoids the risks that some UK companies are excluded from our analysis.

We have created different visualisations of this data to establish whether the UK government should think small first. The data reveals a stark outcome—by far most companies are private companies (99.87%). The vast majority of companies are very new, and the high proportion of new companies means that a significant number have not filed any accounts. There is no data as to account type available for 56.85% of companies on the public register. 40% of companies file the smallest number of accounts, whereas only 2.2% of companies file ‘full’ accounts. These figures alone create a prima facie case for the need to think small first. We then add time into these relationships—when we factor in date of incorporation of companies contained on the current register, we see that companies currently on the register are overwhelmingly new companies. This means that there is limited information about them publicly available, but the information that there is shows newer companies are more likely to file the smallest type of accounts. This demonstrates a trajectory to need to think small first more rather than less.

One interesting qualification to the need to think small first is in respect of mortgages. Here, whilst more mortgages have been granted by all private companies in aggregate than by all public companies in aggregate, we see public companies (traditionally viewed as larger companies) granting more mortgages per company on average over their life. As would be expected, older companies have granted more mortgages than younger ones. However, companies whose account types are currently reflective of bigger financial metrics have granted more total mortgages per company than those with account types reflective of smaller financial metrics. The same is true when we only review live mortgages. Thus, we can say that thinking small first is important for company law, and in aggregate the grant of mortgages by smaller companies is important, but for any individual company, mortgage law needs to think big first. Thus, mortgage law needs to cater for both smaller companies—which have granted most mortgages by number—and larger companies, which each grant more mortgages than smaller companies. As such, company law should think small first generally, but also needs to cater for big companies when exploring the grant of mortgages. These two insights have profound policy implications for the future regulation of companies within the UK.

Thinking small first has always been argued for on intuition and rough figures from approximately twenty years ago. This article demonstrates, using empirical techniques novel to corporate law, a pressing modern need to think small first in all aspects of corporate law analysis. To an extent, a large number of our insights will be already intuitively accepted as being the case. This empirical evidence verifying such intuitions, though, is important. Indeed, it has been stated that the main purposes of such empirical legal research is to test such intuitions^25^ and verify such hunches.^26^

There are five main implications of the novel, holistic empirical research deployed in this article. First, a large amount of corporate law analysis focuses on larger companies: analysis tends to focus on matters relevant for companies whose shares are listed on the public stock exchange. There could be a number of justifications for such focus, including economic or social impact, availability of information, natural fit to dominant agency cost analysis, etc.^27^ This article does not argue that such a focus is incorrect, merely that the quantitative analysis of the UK’s corporate database seems to suggest that smaller companies are dominant in the corporate landscape. The outcome of this article’s study means that analytical focus on larger companies needs to be clarified and justified, or replaced. As such, this article challenges the fundamental conventional wisdom underpinning what is important in corporate law research. Second, most companies are newer and smaller companies, and we lack financial information about such companies. We propose event-driven filings to remedy this information gap which is major across the UK corporate landscape, and material in respect of corporate contracting. Third, we entirely lack formal corporate governance advice for smaller companies. Existing tools are available, but need to be repurposed to be applicable. We outline certain considerations that such advice will need to cater for in Sect. 4.3. The scale of the problem identified by this article demonstrates that such corporate governance guidance/codes are urgently required within the UK. Fourth, the UK’s accounting framework manages to attain a ‘think small first’ approach in substance. However, the form that it uses to do so involves stating the rules that apply to larger companies, then providing carve-outs for smaller companies. We need to ‘think small first’ in form as well as substance to ensure that the compliance burden in *understanding* such rules lands where most suitable and appropriate. This suggests a major change is required in respect of the form of UK corporate legislation. Fifth, a major exception to this appears to be corporate finance: larger companies grant more security per company than smaller companies. Whilst the aggregate of the smaller companies is larger than the aggregate of the larger companies, that any individual larger company is more likely to grant security than any individual smaller company remains important for corporate finance debates and an insight of great relevance to the foregoing arguments.

This paper proceeds as follows. Section 2 outlines the empirical methodology that we deployed. Section 3 outlines the results of our study. Section 4 outlines the implications of our findings, and Sect. 5 concludes.

## Empirical Methodology

### Data and Base Methodology

Our data was downloaded from the UK’s public registry, Companies House.^28^ UK companies only exist when incorporated by the registrar of companies,^29^ and therefore this sample will include all UK companies. Companies can leave the UK register in a number of ways. First, they can be struck off the register by the registrar if the latter considers that the company is not carrying on any business.^30^ Second, they can be voluntarily struck off by the company’s directors if the directors have given notice to potential creditors and if the company has not done anything for three months.^31^ Third, they can be removed by an insolvency practitioner following the conclusion of a liquidation process.^32^ As such, the UK corporate register is, for our purposes, constitutive—you must be on the register to be a UK company, and defunct entities are removed from the register. This is different to the position faced in respect of the limited partnership, a registered vehicle of UK partnership law, which currently does not allow for defunct entities to be removed from the register.^33^ Thus, the snapshot we used contained data in respect of all UK companies as at the relevant date, and no defunct entities are included in our data.

The precise data science techniques that we applied to the data are available freely online.^34^ As noted above, the challenges were in scale rather than method, and so the methods that we deployed were mostly quite simple. We mainly used Python’s Pandas library via a Google Colaboratory notebook. We worked with basic SQL queries: selecting rows, joining groups, adding features, and counting features.

Within the dataset, then, we first explored our ‘static variables’. These are our core variables—the main issues that we wish to explore. Thus, our static variables are basic splits of how many companies fall within each category within the static variable. In addition to exploring these static variables across our dataset, we wanted to see whether these variables were consistent when time was factored in: is the split within each static variable uniform, or does it vary if we factor in company age.

### Static Variables

In this section we review the methodology and underlying law behind our static variables. Here, we explore type of vehicle, and account type. Mortgage information is separately discussed later. In each part, we outline the information that is available in our datasets and the law underpinning the area, including the extent to which the information can be stated to be accurate.

#### Type of Vehicle

The first static variable we explored was company type. There are a number of vehicle types that are registered on the UK public register. First, private companies are the default company vehicle within UK company law.^35^ Within this category, we have private companies with limited liability and unlimited liability for shareholders.^36^ Within the category of private companies with limited liability for shareholders, such liability can be limited by shares or by a guarantee from the shareholders.^37^ A private company with limited liability for its shareholders must have the word ‘limited’ after its name,^38^ unless it exists for charitable purposes, in which case it can obtain an exemption.^39^ Second, public limited companies^40^ are those which are able to offer their shares to the public,^41^ although public limited companies do not have to do so.^42^ Thus, private companies are unable to offer their shares to the public, or allot any shares with a view to them being offered to the public—so cannot have their shares admitted to a stock exchange.^43^ Third, community interest companies are those that have to undertake some form of community interest project rather than being purely profit seeking.^44^ Fourth, limited liability partnerships are vehicles primarily intended for professional services firms to allow, effectively, a large partnership vehicle with limited liability for all of its partners.^45^ Fifth, limited partnerships are primarily investment vehicles which allow passive investors to obtain limited liability, but not all partners.^46^ Within this category, a new subset of ‘professional fund limited partnership’ was created to facilitate professional investment in the limited partnership form.^47^

Various other types of corporate vehicle are included in the UK corporate database. As such, the full list of types of vehicles provided in Companies House information is therefore included in the categories listed in Table 1, together with whether they are included or excluded from the majority of our analysis (once we have noted how many of each type of company are on the public register, and how the excluded companies may impact on other aspects of analysis).

**Table 1:** Type of companies included on the public register

Company type	Description
*Charitable Incorporated Organisation*	As described above. Excluded from majority of analysis as predominantly charitable
*Community Interest Company*	As described above. Excluded from majority of analysis as predominantly charitable
*Converted/Closed*	According to Companies House, this means that the entity has been removed from the register, and is therefore excluded from the majority of our analysis^a^
*Further Education and Sixth Form College Corps*	These are educational establishments incorporated under a specific education act.^b^ They must be a charity,^c^ and are therefore excluded from the majority of our analysis
*Industrial and Provident Society*	Such societies have a long history of being incorporated under separate legislation.^d^ Companies House does not hold information on such companies,^e^ they are therefore excluded from the majority of our analysis
*Investment Company with Variable Capital*	Investment companies with variable capital are a form of open-ended investment company.^f^ They do not have to file any information with Companies House,^g^ and so are excluded from the majority of our analysis
*Investment Company with Variable Capital (Securities)*	As above
*Investment Company with Variable Capital (Umbrella)*	As above
*Limited Liability Partnership*	As described above. Excluded from majority of analysis as this is a partnership vehicle rather than a corporate vehicle
*Limited Partnership*	As described above. Excluded from majority of analysis as this is a partnership vehicle rather than a corporate vehicle
Old Public Company	This type of company refers to those public companies incorporated prior to 1980, for which a special transitional arrangement was entered into when the Companies Act 1980 was introduced.^h^ They are thus included as public companies in our analysis
*Other Company Type*	This appears to be the category into which Companies House places legal forms which do not fully fit within other categorisations. As they do not evidently relate to public or private companies, we have excluded this category from the majority of our analysis
*Other company type*	As above
*PRI/LBG/NSC (Private, Limited by guarantee, no share capital, use of 'Limited' exemption)*	As described above. Excluded from majority of analysis as predominantly charitable
*PRI/LTD BY GUAR/NSC (Private, limited by guarantee, no share capital)*	As described above. Excluded from majority of analysis as predominantly charitable
*PRIV LTD SECT. 30 (Private limited company, section 30 of the Companies Act)*	This refers to an historic statute which provided the equivalent of the modern ability for charitable companies to avoid having to use the word ‘limited’.^i^ In line with the approach for the modern equivalent, it is excluded from the majority of analysis.
Private Limited Company	As described above. Included in our analysis
*Private Unlimited*	As described above. Excluded from majority of analysis as lacking limited liability
*Private Unlimited Company*	As described above. Excluded from majority of analysis as lacking limited liability
*Protected Cell Company*	The protected cell company is a special type of investment vehicle.^j^ They are registered^k^ with the Financial Conduct Authority, which maintains the primary register for this vehicle.^l^ As such, they are excluded from the majority of our analysis
Public Limited Company	As described above. Included in our analysis
*Registered Society*	These fall under the same regime as industrial and provident societies, and are mostly excluded accordingly
*Royal Charter Company*	Royal Chartered Companies are chartered by the Crown. Their members have no liability for the debts of the company.^m^ This method has largely been supplanted and used for only charitable purposes,^n^ and so is excluded from the majority of our analysis
*Scottish Charitable Incorporated Organisation*	This is a specific form of charitable organisation, and is therefore excluded from the majority of our analysis^o^
*Scottish Partnership*	This is a form of partnership vehicle which has to register with Companies House if all of its partners are companies.^p^ As it is a partnership vehicle it is excluded from the majority of our analysis
*United Kingdom Economic Interest Grouping*	This concept originated under EU law,^q^ but each vehicle under this regime registered with Companies House was converted to a UK specific vehicle as part of the Brexit process.^r^ They provide no limited liability for members and appear as a hybrid between a partnership and corporate vehicle,^s^ and are therefore excluded from the majority of our analysis
*United Kingdom Societas*	This concept also originated under EU law,^t^ but each vehicle under this regime registered with Companies House was converted to a UK specific vehicle as part of the Brexit process.^u^ As these entities are UK recognitions of European entities, they are excluded from the majority of our analysis

^a^https://wck2.companieshouse.gov.uk/goWCK/help/en/stdwc/company_H.html (accessed 25 November 2021)

^b^Further and Higher Education Act 1992 s. 16

^c^Ibid., s. 22A

^d^Industrial and Provident Societies Act 1965. See discussion in Cross (2008). They are now incorporated under the Co-operative and Community Benefit Societies Act 2014

^e^https://wck2.companieshouse.gov.uk/goWCK/help/en/stdwc/excl_ch.html (accessed 25 November 2021)

^f^Financial Services and Markets Act 2000, s. 236; Open-Ended Investment Companies Regulations 2001 (SI 2001/1228). See Hudson (2000)

^g^https://wck2.companieshouse.gov.uk/goWCK/help/en/stdwc/excl_ch.html (accessed 25 November 2021)

^h^Companies Act 1980, s. 8

^i^Companies Act 1985, s. 30

^j^Risk Transformation Regulations 2017 (SI 2017/1212)

^k^Ibid., Regulation 21

^l^Ibid., Regulation 32

^m^*Elve v Boyton* [1891] 1 Ch. 501 CA

^n^See Davies et al. (2021), paras. 1-006–1-012

^o^Charities and Trustee Investment (Scotland) Act 2005. See Ford (2006).

^p^Partnerships (Accounts) Regulations 2008 (SI 2008/569); Scottish Partnerships (Register of People with Significant Control) Regulations 2017 (SI 2017/694). See McKenzie-Skene (2017)

^q^European Economic Interest Groupings Regulations 1989 (SI 1989/638). See Burnside (1990)

^r^European Economic Interest Grouping (Amendment) (EU Exit) Regulations 2018 (SI 2018/1299)

^s^Morse et al. (2021), para. 1.228

^t^Regulation 2157/2001 on the Statute for a European Company [2001] OJ L294/1

^u^European Public Limited-Liability Company (Amendment etc.) (EU Exit) Regulations 2018 (SI 2018/1298)

In this article we are concerned with whether we are right to think small first for profit seeking companies. Unlimited companies, companies limited by guarantee, community interest companies, and those exempt from using the word ‘limited’ are associated with charitable, non-profit seeking ventures.^48^ Such vehicles have different conceptual considerations to profit seeking companies,^49^ and partnership vehicles also have different conceptual considerations.^50^ We therefore excluded such charitable companies and partnership vehicles from the majority of our analysis, along with other vehicles noted in Table 1.

As such, we removed those entries in Table 1 in italics from most of our statistical analysis, and mostly limited the scope of our study to private companies limited by shares and public companies. Companies can be re-registered into different types: from private to public,^51^ from public to private,^52^ from limited to unlimited and vice versa.^53^ In each case, though, the change is effected upon the registrar of companies processing the change.^54^ Unless a re-registration has been processed by the registrar, it will not have legally occurred. We can therefore state that the data contained within our dataset on this point is definitive. It is generally presumed that public companies will be larger than private companies.^55^ There are exceptions to this,^56^ such as industries which strategically utilise private companies^57^ and large private companies.^58^ As such, we can state that whether a company is public or private is a rough proxy for company size, but not a perfect one. A preponderance of private companies is prima facie evidence of the need to think small first, but not conclusive of a requirement to do so.

Different legal regimes apply in the UK for (a) a private company, (b) a public company whose shares are not listed, and (c) a public company whose shares are listed. Certain legal restrictions apply to public companies (whether or not their shares are listed) and not private companies: such as minimum levels of share capital,^59^ additional requirements for meetings,^60^ and further restrictions on interactions between the company and directors.^61^ It is not the case that all plcs are listed,^62^ but if they are listed then a further set of legal restrictions apply, such as governance requirements (including requiring a measure of independence in directors).^63^ As such, the legal regime applicable across public and private companies, and across listed and unlisted companies, differs. Similarly a number of non-legal considerations which frequently underpin legal analysis are inherent within this distinction. Thus, if shares are freely tradable a market for corporate control develops,^64^ which in turn disciplines directors.^65^ Neither of these are present without a public market for shares, meaning that this disciplinary function does not apply to private companies.^66^ Further, the presence of a capital market is often used to justify limited liability for shareholders,^67^ and underpins the ‘master problem for research’^68^ in corporate law—the ‘folklore’^69^ of the separation of ownership and control in large US public companies.^70^ We can therefore note that theoretical conceptions of private companies are different from those of their listed counterparts. Whilst we cannot state that all plcs are listed, we can state that all private companies are not.^71^ A preponderance of private companies would therefore demonstrate that these theoretical considerations linked to a capital market applied to relatively few companies rather than acting as the base level of analysis for corporate law.

This is, of course, a negative conception—we display concepts where companies are ‘not listed’ rather than hold a positive understanding of concepts applicable to private companies. This is the traditional approach taken to conceptual issues arising in respect of non-listed companies. Wells stated of the overlapping concept of a close company that it is

easy to grasp but hard to define. Although a bundle of special features can be cited as marks of a close corporation, the close corporation is most easily understood as a legal entity when contrasted to what it is not – the large corporation whose shares are publicly traded (the ‘public corporation’).^72^

There is an overlap between a private company and what commentators refer to as a ‘close’ company. It has been argued that a ‘close’ company is merely a bastardisation of ‘closed’ company,^73^ which is used in economics in contrast to a company which is listed.^74^ The precise delineation of what is and is not a close company is very unclear, but the literature tends to use close and private synonymously.^75^ Given the indeterminacy of what is and is not a close company, it is fair to say that not all private companies are close companies. Clarification of this is outside the scope of this article: for now, we follow the approach taken by other commentators of using private and close companies synonymously.

Features normally associated with close, or private, companies can be identified. First, there is likely to be a small number of shareholders, normally with most involved in management.^76^ This involvement could be as directors, employees, a combination of both, or even other constituencies which are traditionally seen as ‘external’ to the company—such as creditor or landlord.^77^ Second, the business is likely to be a major source of income for those who are involved in management, albeit that this income could be taken in any capacity.^78^ Third, the operations of a private company (in its widest sense: including internal governance and external relations) are likely to be less sophisticated than we see in public companies.^79^ Fourth, certain shareholders are likely to be dominant within the company.^80^ As such, it has been argued that most private companies should be seen conceptually as similar to partnerships.^81^ However, as they use the corporate form, there are differences in legal structure: primarily that the minority cannot withdraw funds from the corporate vehicle at will, thus making it not a perfect conceptualisation.^82^ It has thus been argued that corporate participants require more protection in private and close companies than they do in listed companies or partnerships.^83^

Courts do sometimes provide such protection—with certain company law protections applying almost exclusively in the context of private (or, at least, unlisted) settings,^84^ leading some to argue that there is already a separate law that governs the law of closed companies.^85^ Whilst corporate law at times recognises this difference, corporate governance is weak at doing so—with corporate governance guidance being almost exclusively aimed at larger, listed companies.^86^ This is not to say, of course, that legal tools which are used to regulate the behaviour of listed companies cannot be used to regulate behaviour in private companies, but the precise way in which such regulation is deployed may well vary.^87^

#### Account Type

Our second static variable is the type of accounts filed by each company. The UK has historically^88^ required the filing of a company’s accounts with the registrar of companies, which reflects the historic emphasis on public disclosure rather than private verification.^89^ These accounts relate to a particular financial period, usually of 12 months,^90^ but this can be shortened to 6 months or extended to 18 months.^91^ Accounts must be publicly filed within a timescale following the end of the period to which those accounts relate. This is normally 6 months for a public company and 9 months for a private company.^92^ There is thus an inevitable delay in publicly available accounting information—it inevitably relates to a historic period, and is inevitably published after the end of that historic period.

Company accounts must, at their core, present a true and fair view of the company’s financial position, and contain a profit and loss account and a balance sheet in respect of the company.^93^ The company’s accounts are used as the basis to establish if it is able to make distributions,^94^ for corporation tax purposes^95^ and to inform investors.^96^ Their importance means that they must usually be audited by professional accountants,^97^ and must also frequently include a number of additional requirements.

The precise scope of what must be filed by each company, though, varies based on primarily financial characteristics, with bigger companies having to file more. There are four basic categories of companies for accounting purposes: micro, small, medium and full. A company must file full accounts unless it meets a test to fall within a smaller category.^98^ To fall within a smaller category, the company must, within the period, meet two of three criteria. The three criteria are that the company’s turnover falls within certain boundaries, that its gross asset value (referred to as a company’s balance sheet size) falls within certain boundaries, and that the number of employees falls within certain boundaries. To be medium sized, it must have two out of the three of turnover not exceeding £36m, a balance sheet total not exceeding £18m and no more than 250 employees. Were this division merely binary it would be relatively simple.^99^ However, further complications arise when we factor in the other two categories, thus a company falls within the small company regime if it meets two of the three of turnover not exceeding £10.2m, a balance sheet total not exceeding £5.1m, and no more than 50 employees. A public company cannot qualify as a small company.^100^ To fall within the micro company regime, it must meet two out of the three of turnover not exceeding £632,000, a balance sheet total not exceeding £316,000, and the number of employees not exceeding 10.^101^ A public company cannot be a micro-entity.^102^

The precise level of detail that requires to be filed for each account category varies.^103^ Additional complications arise, too. First, where multiple companies are within the same corporate group,^104^ they must consolidate the accounts of the group into one set of group accounts.^105^ Here, Companies House notes that the top company within the consolidated group has ‘group’ accounts, and the subsidiaries have ‘subsidiary’ accounts. Second, if for any particular period a company has no significant accounting transaction then it is classified as dormant.^106^ A company which has been dormant for a whole year and which is a subsidiary of another UK company does not have to prepare or file financial statements,^107^ subject to certain conditions (such as the parent guaranteeing any outstanding liabilities of the subsidiary).^108^ Third, small or micro companies may opt to provide even less detail in their accounts (they can simplify their profit and loss account, balance sheet, or both), known as ‘abridged’ accounts, if shareholders unanimously so elect.^109^ Fourth, small companies can also be exempt from requirements to have their accounts audited by external accounting professionals.^110^ As such, abridged accounts can be audited or unaudited. Fifth, we note above that accounts are used for distributions, and that there is an inherently historic element to corporate accounts. If a company wants to declare a dividend before its complete financial statements are available, it can prepare initial accounts, a short-form version of accounts to provide directors with sufficient evidence that the company has sufficient to pay distributions.^111^

Companies House classifies a company’s accounts based on the most recent version filed. Thus, the Companies House categories of account type, together with an explanation, are set out in Table 2. To simplify matters, we have also grouped accounts into ‘smallest’ (applicable for the smallest companies), ‘subsidiary’ (for those evidently the subsidiary within a consolidated group), ‘full’ (for the largest companies and the parent of consolidated groups), and ‘no detail available’ (for companies where no detail has been filed, or only interim accounts have been filed). It should be noted that a company can file accounts at a higher standard than is required. Our concern, though, is in identifying the level of accounts that are *required* to be filed.

**Table 2:** Account type

Account type	Description	Grouping
Dormant	Falls within the dormant regime described in text	Smallest
Audited abridged	Accounts are audited, capable of being abridged and are abridged under the regime described in text	Smallest
Audited unabridged	Accounts are audited, capable of being abridged but are not abridged under the regime described in text	Smallest
Small	Falls within the small regime outlined above	Smallest
Total exemption full	The company is entitled to an exemption under the regime outlined above, but chooses to comply with higher accounting standards	Smallest
Total exemption small	As above	Smallest
Medium	Falls within the medium regime described in text	Smallest
Partial exemption	A partial exemption is obtained under the regime outlined above	Smallest
Filing exemption subsidiary	Relates to a subsidiary company which has adopted the filing exemption regime outlined above	Subsidiary
Audit exemption subsidiary	Relates to a subsidiary company which has adopted the audit exemption regime outlined above	Subsidiary
Full	Falls within the full accounts regime described in text	Full
Group	Falls within the group regime described in text	Full
Accounts type not available	Companies House leaves this field blank, indicating that no account information is available	Complete picture unavailable
No accounts filed	Companies House notes this company as having filed no accounts yet	Complete picture unavailable
Initial	Initial accounts have been filed under the regime described in text	Complete picture unavailable

In terms of evaluating the need to think small first, accounts are primarily based on certain financial metrics, and therefore are a good way to establish this need. There is a legal obligation that accounts maintained and filed must provide a ‘true and fair view’ of the financial position of the company.^112^ The information is, however, intrinsically historic, meaning that the information provided can be out of date very quickly. For example, in respect of the collapse of UK construction company Carillion, a UK parliamentary inquiry noted:

Carillion’s collapse was sudden and from a publicly-stated position of strength. The company’s 2016 accounts, published on 1 March 2017, presented a rosy picture. On the back of those results, it paid a record dividend of £79 million—£55 million of which was paid on 10 June 2017. It also awarded large performance bonuses to senior executives. On 10 July 2017, just four months after the accounts were published, the company announced a reduction of £845 million in the value of its contracts in a profit warning. This was increased to £1,045 million in September 2017, the company’s previous seven years’ profits combined. Carillion went into liquidation in January 2018 with liabilities of nearly £7 billion and just £29 million in cash.^113^

In addition, it has been argued that the newer a company is, the more likely it is to fail.^114^ However, for a private company, its first accounting period will, by default, end on the date falling on the 1 year anniversary of its incorporation,^115^ with its accounts needing to be filed within 9 months of that date.^116^ This means that public information about a new private company may not be available for up to 21 months after it has been incorporated, and that time can be extended.^117^ This represents a considerable time lag for the public provision of financial information.

Accounts, or confirmation of their relevant exemption, must be filed at Companies House within the applicable time periods,^118^ otherwise the company is subject to a fine^119^ (which is rigidly enforced^120^) and could be struck off.^121^ This means that the data filed at Companies House, whilst historic, will be complete. As such, our static variables are company type which is definitive in status but merely indicative of a think small first requirement; and account type which, being so heavily based on financial metrics, is conclusive evidence of any requirement for a think small first mentality but is inherently historic and so not definitive as at the date it is filed. Each metric on its own is a strong indicator of any requirement to think small first. Neither can be stated to be conclusive, though. This does not matter—LoPucki noted that legal empirical research verifies hunches that already exist. Accordingly, it does not require the same statistical rigour that is required in other social science quantitative empirical research.^122^ As such, that our two metrics provide a strong indication towards any need to think small first, but cannot conclusively prove it, does not detract from their utility as proxies for the issue. When coupled together, their values as proxies are multiplied.

#### Interaction of Static Variables

Each static variable will, of course, be important on its own. It will tell us the number of vehicles on the UK public register within each company type and account type. However, when we apply multiple static variables, we will see themes running within company types. Thus, we will explore interaction between company type and accounts—whether there is a correlation between certain types of companies and account types.

### Factoring in Time

The foregoing provides insight into the snapshot of the UK corporate register that we have obtained. However, the data can provide further information as to whether the calibrations identified apply uniformly across all companies. The dataset provides the date of incorporation of the company, being the date that the corporate registrar processed the incorporation form for the company,^123^ undertaking the ‘mysterious rite’^124^ of creating a new person. We can thus explore whether the variables outlined above are consistent across different ages of companies, or whether there are differences correlating to company age. This lets us test the general assumption operating in business life, which is that companies start small and generally grow over time if they do not enter insolvency.^125^ We can therefore apply time series analysis to the static variables outlined above, to see whether there are any changes to calibrations that apply over time.

It should be noted that the data that we have only relates to the age of companies on the database as at a given time. Companies can leave the corporate register,^126^ and any companies which have left the register prior to the date of our dataset are not reflected in it. We cannot use our data to explore, for example, whether more companies are being incorporated now than were previously incorporated, only whether current companies are mostly older or younger.

### Corporate Finance: Mortgages

Our final variable is the number of mortgages granted by the company. We explored this as a separate and stand-alone variable to ease data presentation challenges—including it as a third static variable created too many inter-relations and confused the presentation of results. Mortgage has a technical meaning under English law,^127^ but its use in Companies House data is much broader: every time a company grants a charge,^128^ mortgage or other real right in security (other than a pledge)^129^ it must register that at Companies House.

The Companies House data in respect of mortgages therefore represents all real rights in security (fixed or floating), or security over the company’s tangible and intangible property, that have been granted by the company in question, other than a pledge.^130^ It is therefore an important metric in respect of corporate finance. The ability to grant security has been argued to be economically efficient,^131^ but this is debated.^132^ For our purposes, the role that security plays in corporate finance is contentious: either being seen as a signal that a lender is willing to provide structural finance to a company,^133^ or a sign of weakness of the borrower (that they could not obtain finance without proffering collateral^134^). Factoring in whether larger or smaller companies are more likely to grant security will provide insights into the operation of this debate within the UK market.

Registration of security must be completed by the later of 21 days following the grant of security^135^ or such other date as may be provided in a court order.^136^ Such an order will only be granted if failure to comply with the initial 21-day time period was accidental or does not prejudice shareholders or creditors of the company or it is otherwise just and equitable to grant the extension.^137^ Even should that apply, it is at the court’s discretion whether such an order be granted.^138^ This means that all parties are incentivised to avoid both the cost and risk inherent in court proceedings by ensuring that the document is registered within 21 days.

Failure to register the document within the relevant timescale means that it is void against an insolvency practitioner or creditor of the company.^139^ As the purpose of such a security right is to provide its holder with an advantage on the insolvency of the granter,^140^ this will be avoided by all parties. As such, whilst there is an inherent time lag between the grant of the security document and its registration, this is smaller than that which exists for accounts, and in a similar way the register will reflect all so registered.

Security can be released by the creditor—for example, it must be released upon repayment of the debt.^141^ The UK regime therefore provides that security can be noted as being satisfied—either wholly or partially.^142^ Registration of satisfaction of charges is voluntary, though, and is not affected by the same time restrictions that affect the registration of charges.^143^ There is therefore a risk that charge registration will be overinclusive—that all grants of security are registered but not all satisfactions are.^144^ This will be partially mitigated by practice—an incoming lender receiving security will request that the company’s record be up to date and so include all satisfactions, for maximum clarity that their security will rank first.^145^

Thus, for this category, we count the number of mortgages granted by each company within each type, including the total of those granted over the life of the company, and the number of those that are outstanding. We then factor time into this variable.

## Results

We thus took the data provided by Companies House as at 1 June 2021 and applied the foregoing methodology to it. A more detailed analysis of the precise steps that we took to achieve those ends is publicly available.^146^ This section sets out the results of our study.

### Static Variables: The Basic Importance of Thinking Small First

#### Company Type

First, we see a total of 4,956,374 vehicles on the UK corporate register in our data. The breakdown of these companies by type is shown in Table 3. The vast majority are private limited companies—4,608,200 (92.98% of the total). Only 6142 (0.12% of the total) are public limited companies. Other research unveils that approximately 1,410 of these have their shares listed on the London Stock Exchange, and only approximately 489 of those are non-investment companies listed on the Main Market of the London Stock Exchange.^147^ This means that just under one quarter of UK plcs have their shares listed in the UK—with over three quarters either not having their shares listed on any public exchange, or having such listing outside of the UK. The focus in this study is on public and private limited companies, but it is worth noting that there are a number of vehicles which considerably outnumber plcs—including limited liability partnerships (52,713, or 1.06% of the total) and limited partnerships (54,809, or 1.11%^148^). As such, there are 8 times as many limited liability partnerships on the UK public register as there are public limited companies.

**Table 3:** Company type

Company type	Number	%
Charitable Incorporated Organisation	25,044	0.51^a^
Community Interest Company	24,642	0.50
Converted/Closed	1	0.00
Further Education and Sixth Form College Corps	2	0.00
Industrial and Provident Society	183	0.00
Investment Company with Variable Capital	624	0.01
Investment Company with Variable Capital (Securities)	10	0.00
Investment Company with Variable Capital (Umbrella)	74	0.00
Limited Liability Partnership	52,713	1.06
Limited Partnership	54,809	1.11
Old Public Company	26	0.00
Other Company Type	4	0.00
Other company type	13,289	0.27
PRI/LBG/NSC (Private, Limited by guarantee, no share capital, use of ‘Limited’ exemption)	40,921	0.83
PRI/LTD BY GUAR/NSC (Private, limited by guarantee, no share capital)	107,028	2.16
PRIV LTD SECT. 30 (Private limited company, section 30 of the Companies Act)	17	0.00
Private Limited Company	4,608,200	92.98
Private Unlimited	120	0.00
Private Unlimited Company	4,307	0.09
Protected Cell Company	5	0.00
Public Limited Company	6,116	0.12
Registered Society	11,801	0.24
Royal Charter Company	878	0.02
Scottish Charitable Incorporated Organisation	5,024	0.10
Scottish Partnership	239	0.00
United Kingdom Economic Interest Grouping	270	0.01
United Kingdom Societas	27	0.00
Total	4,956,374	100.01

^a^Throughout, we give figures to two decimal places. This, of course, will create some rounding discrepancies if aggregating such two decimal place numbers (including looking at totals), and means that categories with less than c250 in them will appear as 0.00

Of course, a listed company may have a large number of wholly owned subsidiaries within its corporate group. The empirical results of this study outlined below taken from the accounting data suggest that such numbers are insufficient to dwarf the number of private companies which do not fall within such a group structure. Even for those that do, each is a separate legal person.^149^ Whilst they may suffer from pressure from their parent companies,^150^ this pressure is fundamentally different from that suffered *by* the parent company, and is more akin to that suffered by a traditional private company with a dominant shareholder.

There are 40,921 (0.83%) private companies limited by guarantee which are exempt from using the word ‘limited’ in their name under the current regime, and 107,028 (2.15%) private companies limited by guarantee which do not use such exception.

In total, 342,032 (6.9% of the total) are excluded from our categories. This reduces the overall number of companies to 4,614,342—of which 99.87% are private limited companies and 0.13% are public companies. This demonstrates an initial prima facie case for the basic importance to think small first upon our first proxy for company size—as the overwhelming majority of UK limited companies are private, prima facie corporate law analysis should focus on private limited companies rather than public limited companies.

#### Account Type

Our second static variable is account type. This is set out in Table 4 for all vehicles, including those otherwise excluded. Here, we see the largest category is those that have not yet filed accounts, at 1,417,766 (28.6% of the total), followed by those for which the Companies House field was blank at 1,402,499 (28.30% of the total), also indicating that no information is available. This is nearly four times the number of excluded entities, which demonstrates that it is not just excluded entities for which this field is blank. The third largest category is those with a total exemption full, at 1,176,999 (23.75% of the total), followed by dormant companies at 575,298 (11.6%).

**Table 4:** Account type

Account type	Number	%
Accounts type not available	4,169	0.08
Audit exemption subsidiary	13,018	0.26
Audited abridged	1,521	0.03
Dormant	575,298	11.61
Filing exemption subsidiary	312	0.01
Full	97,496	1.97
Group	23,992	0.48
Initial	7	0.00
Medium	354	0.01
No accounts filed	1,417,766	28.60
Partial exemption	24	0.00
Small	70,511	1.42
Total exemption full	1,176,999	23.75
Total exemption small	20,110	0.41
Unaudited abridged	152,298	3.07
Field empty	1,402,499	28.30
Total	4,956,374	100

Table 5 shows these account types with excluded entities removed and divided into our groupings. It demonstrates that the accounting position is unavailable for over half of the companies (56.85%) listed on the public registry as at the relevant date. The second largest group was our ‘smallest’ category, with 40.66% of companies falling within the smallest regime. Not all of these will file abridged information—1,176,999 companies (or 23.75%) filed full accounts despite only being required to file small or medium accounts. If we exclude the ‘no detail available’ category, the smallest category represents 94.2% of companies. Only 2.2% of companies were required to file accounts falling within our ‘full’ category. This demonstrates a further prima facie basis for thinking small first across our second proxy—information is not available for most accounts, but of the information available, the vast majority fall within the smallest category.

**Table 5:** Account types by groupings

Group	Number	%
Full	101,504	2.20
Complete picture unavailable	2,623,442	56.85
Smallest	1,876,337	40.66
Subsidiary	13,059	0.28
Total	4,614,342	100

#### Interaction Between Company Type and Account Type

Matters become clearer when we factor in account type and company type. This is outlined in Table 6. The private company figures outline the same rough percentages as set out above (which is unsurprising as they compose so much of the sample). Calibrations of public company accounts, however, are very different. Here we see that the vast majority of public companies (73.69%) require to file accounts which fall into our full category, with 13.82% having no detail, and 12.24% being in the smallest category. This difference is perhaps unsurprising given that public companies cannot fall within some of the smallest account categories. Nevertheless, it provides three important insights. First, information is much more likely to be available for public companies. Second, whilst private companies are very unlikely to be required to file ‘full’ accounts (at 2.1%), public companies are highly likely to (at 73.7%). This demonstrates that the financial profile of the two different company types are different—we are likely to have more private companies with smaller account profiles, and fewer public companies but those companies possibly having larger account profiles. In respect of aggregate numbers, private companies and small account types are dominant because private companies are more likely to have small account types and there are dramatically more private companies. This justifies the use of both metrics as proxies for the need to think small first: private companies are likely to meet the requirements to be able to file less than full accounts, which are themselves ascertained mostly on the basis of financial metrics.

**Table 6:** Account type and company type

Account type	Private number	Private %	Public number	Public %
Full	96,978	2.10	4,526	73.69
Complete picture unavailable	2,622,593	56.91	849	13.82
Smallest	1,875,585	40.70	752	12.24
Subsidiary	13,044	0.28	15	0.24
Total	4,608,200	99.99	6,142	99.99

### The Temporal Need to Think Small First

**Fig. 1:**
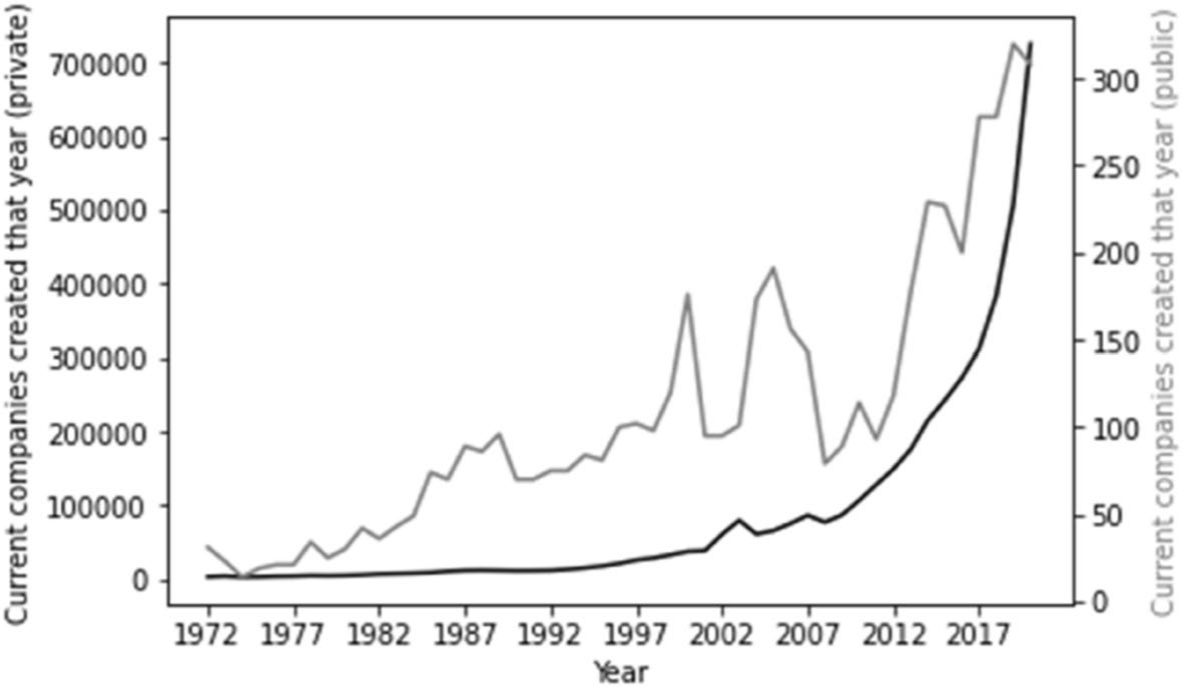
Time series for company type. Our time series graphs for company type commence as of 1972 and for account type commence as of 2000. Older companies, of course, exist, but reflect the exponential trends outlined in these graphs and therefore risk adding no additional information but take up space on the X axis. We therefore adopted this approach to maximise clarity of visualisation of trends applicable to the whole dataset.

Factoring time series into the foregoing metric presents a clear picture. The position in respect of company type is outlined in Fig. 1. Here, we can see that the vast majority of companies on the register have been added recently. For private companies, there is an almost exponential relationship between number of companies and more recent years of incorporation. Given the smaller number of public companies the relationship is less smooth, but follows the same general trend. There are two possible interpretations: either the typical life of a company is very short—thus in every year there is a large number of companies joining the register and then leaving it, such that a snapshot at any time would reflect this—or the number of companies being incorporated is exponentially increasing. In either event, the majority of both private and public companies are new vehicles.

**Fig. 2:**
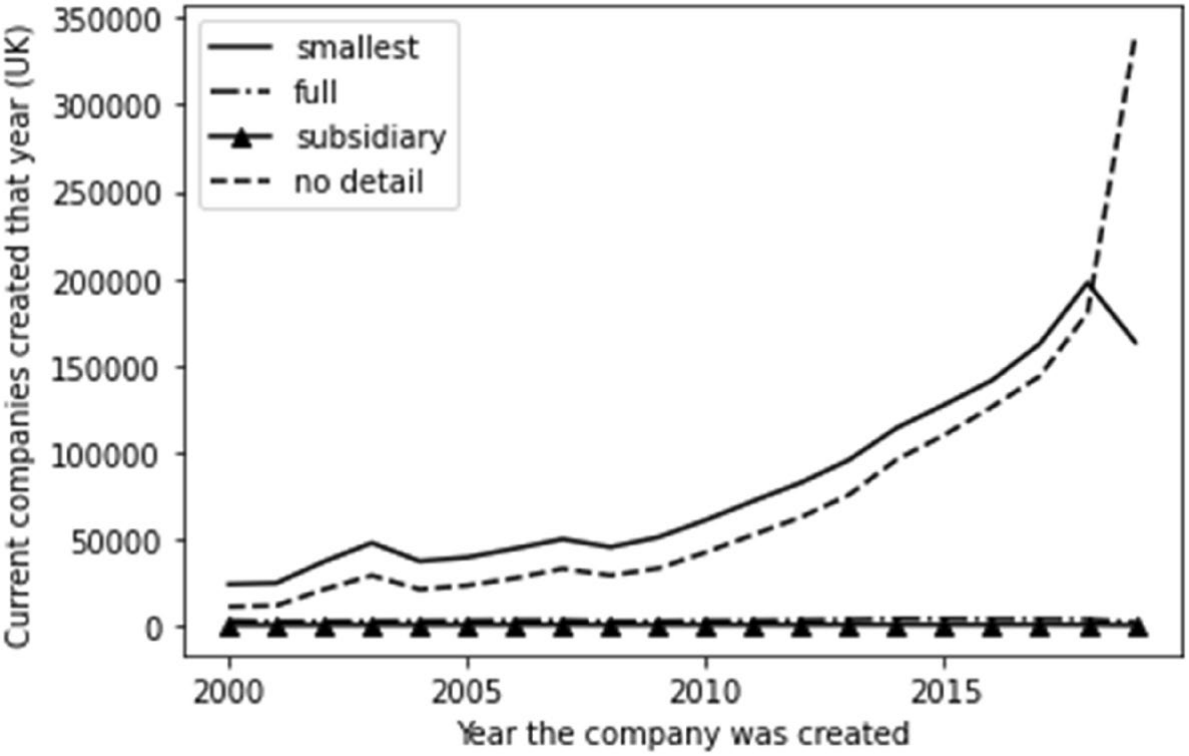
Time series for account type

We also factored time into the company account detail, and the results are set out in Fig. 2. Here, given the scale, we see our groups of subsidiary accounts and full accounts as effectively flat along the X axis. We see a similar exponential growth for ‘no detail available’ as noted for company incorporation, which indicates that there is an overlap between the lack of accounting information and new companies. We do see a growth in the ‘smallest’ category with a sudden drop-off in 2019 which could be explained by the time lag inherent in the preparation and filing of accounts—it may be that a number of the ‘no detail’ category will, upon filing their first historic accounts, be proved to be in different categories—and extrapolating from the current data, this would seem to mostly be the smallest category.

Factoring time into each of the static categories demonstrates that the need to think small first is affected by a temporal factor—any given company on the register is likely to be a newer company, and it is also unlikely to have account information available.

**Fig. 3:**
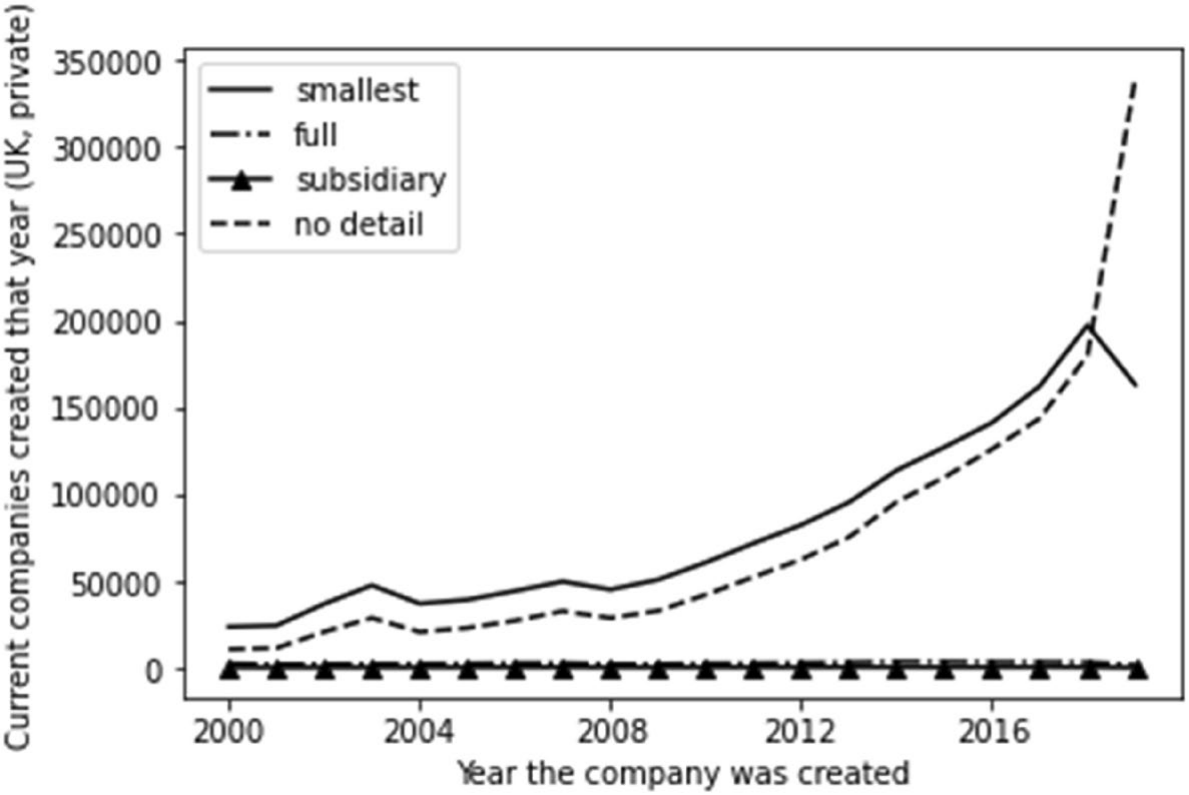
Time series for private company account type

**Fig. 4:**
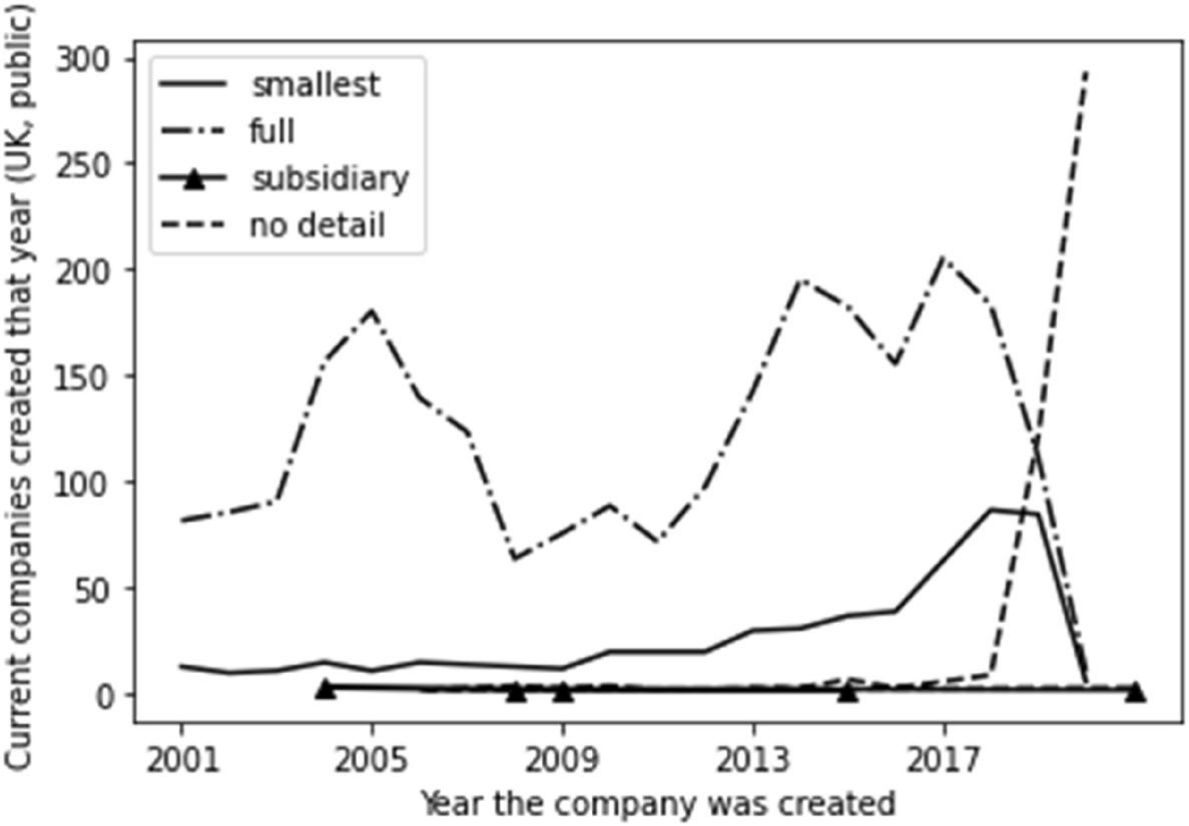
Time series for public company account type

We have also factored time into the breakdown of account type per type of company. Whilst, as would be expected given the numbers involved, the position for private companies (outlined in Fig. 3) reflects the overall picture outlined above, the position for the account type by year of incorporation for public companies is very different. As outlined in Fig. 4, we see public companies consistently being in the full account type. As would be expected, these dramatically reduce for more recent companies and are replaced with ‘no detail available’. This reflects the time lag inherent in filing accounts, although it is more sudden than in the case of private companies. This implies that ‘no detail available’ for public companies is merely down to the inevitable time lag, whereas it is not for private companies.

### Corporate Finance and Thinking Small First

The one area which provides a mixed picture as to the need to think small first is in respect of the number of mortgages granted, indicative of corporate finance. First, we have prima facie evidence of the need to think small first as most of the total number of mortgages were granted by small companies in our proxies for size. Thus, out of a total of 2,754,715 mortgages granted in total across our sample, 2,718,436 (98.68%) were granted by private companies, with 36,279 (1.3%) granted by public companies.^151^ For those mortgages still listed as live, the figures are similarly large at 1,539,662 (99.1%) live mortgages granted by private companies, and 13,951 (0.9%) granted by public companies. Similarly, across our account type, we see 1,667,052 (59.36%) total mortgages granted by companies in our smallest category, 454,462 (16.18%) total mortgages granted by those with no information available, with 643,166 (22.9%) granted by those in the fullest accounts category*.* In respect of those mortgages currently listed as live, we see roughly similar proportions: 922,855 (59.4%) live mortgages granted by companies in our smallest category, 315,706 (20.32%) live mortgages granted by those with no information available, and 302,384 (19.46%) granted by those in the fullest accounts category. As such, it seems that we have prima facie evidence of the need to think small first when it comes to mortgages and security rights as well.

**Fig. 5:**
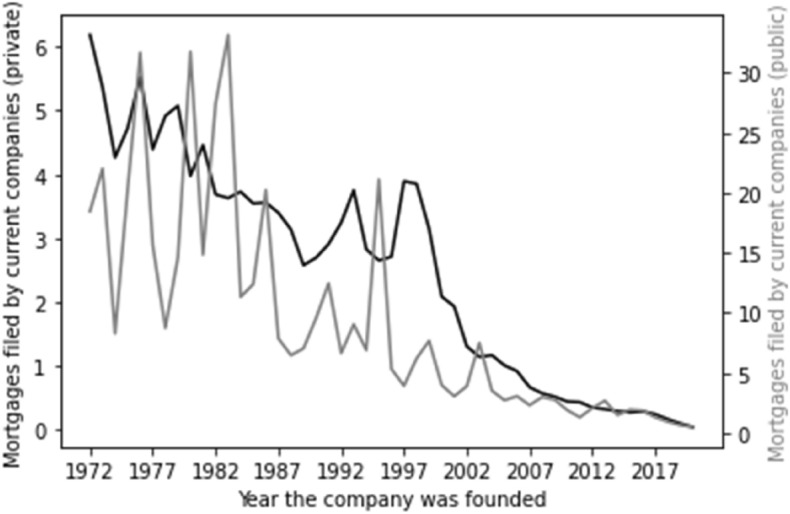
Total mortgages by company age

There is, though, evidence that larger companies grant more mortgages per company. Figure 5 demonstrates the average total number of mortgages granted by public and private companies based on their year of incorporation. Here we see a general trend—older companies have granted many more mortgages than newer companies. The scale, though, varies for private and public companies—we see that public companies granted many more mortgages than private companies on average—with the average for public companies topping 30 and the average for private companies hitting 6. This shows that mortgages are more frequently granted per company by companies falling within our proxy for large companies than they are for our proxy for smaller companies.

**Fig. 6:**
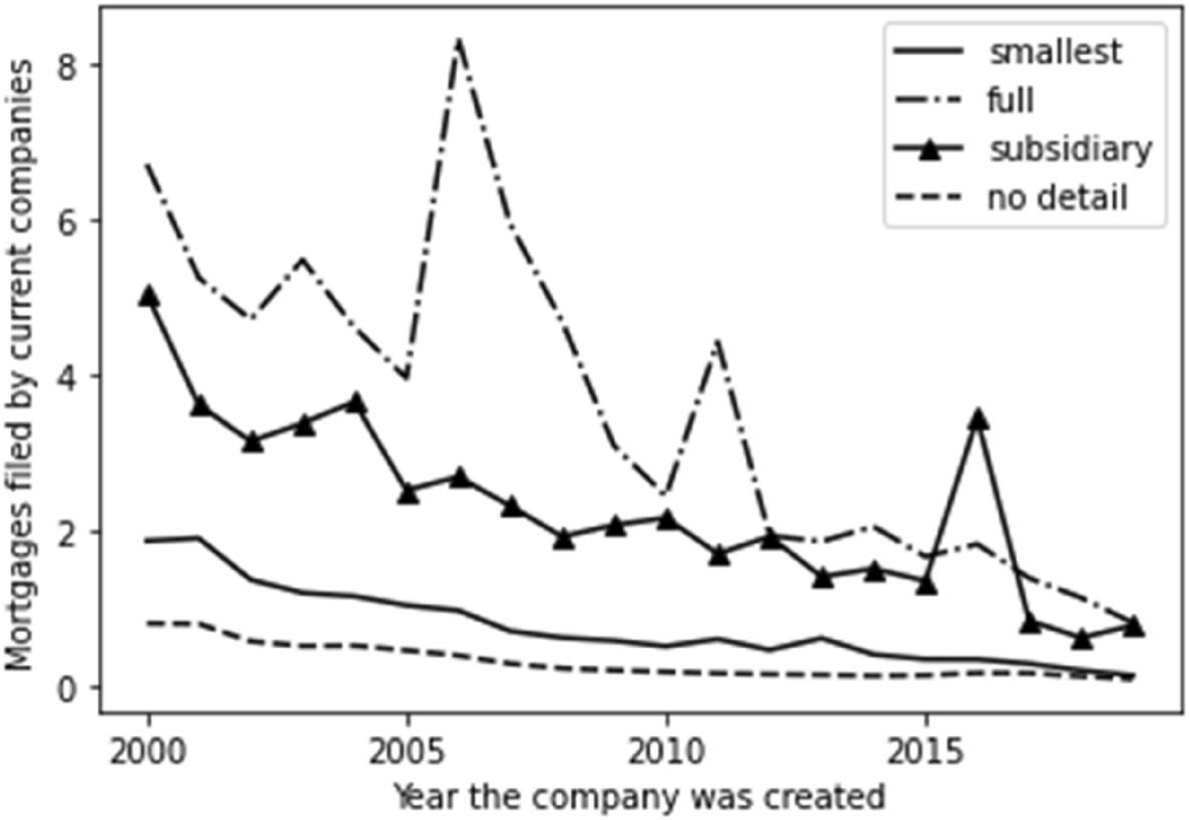
Total mortgages by account type

The same conclusion is reached by exploring the number of average total mortgages by account type, set out in Fig. 6. Again, we see older companies having granted more mortgages than younger companies on average. We also see, though, those filing the fullest accounts grant consistently more mortgages per company on average than those in other account types.

**Fig. 7:**
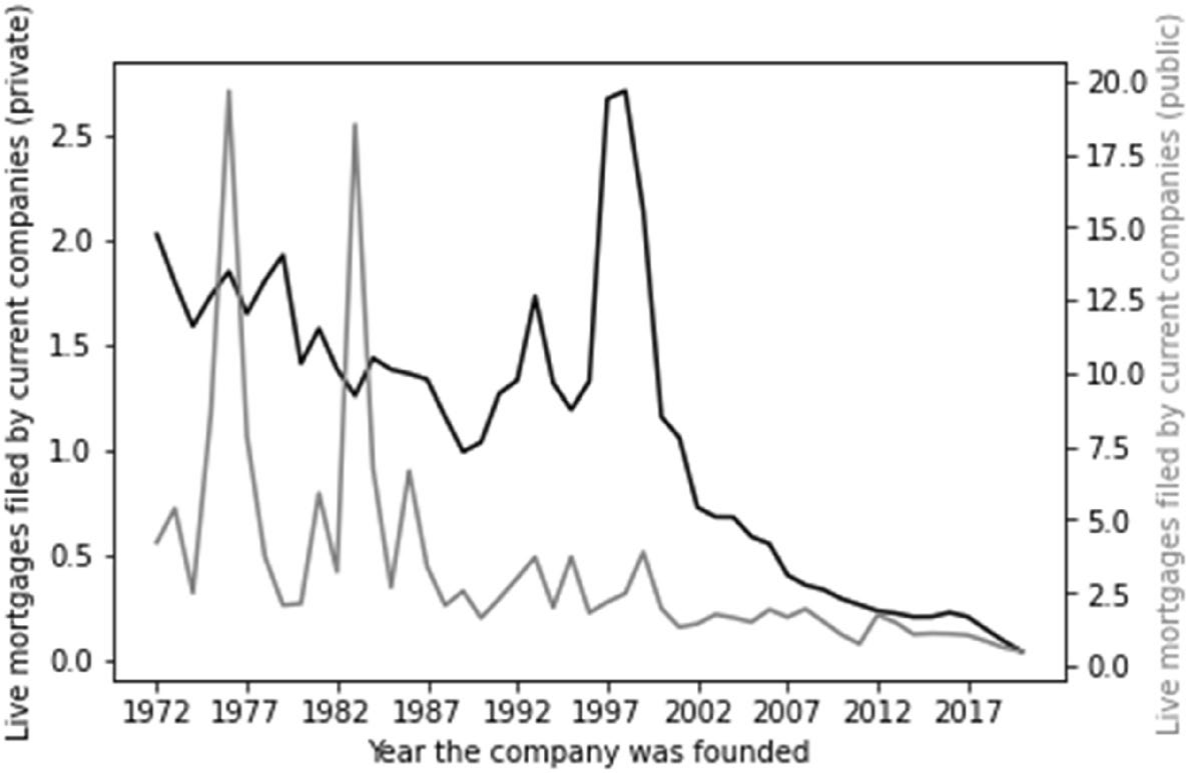
Live mortgages by company type

**Fig. 8:**
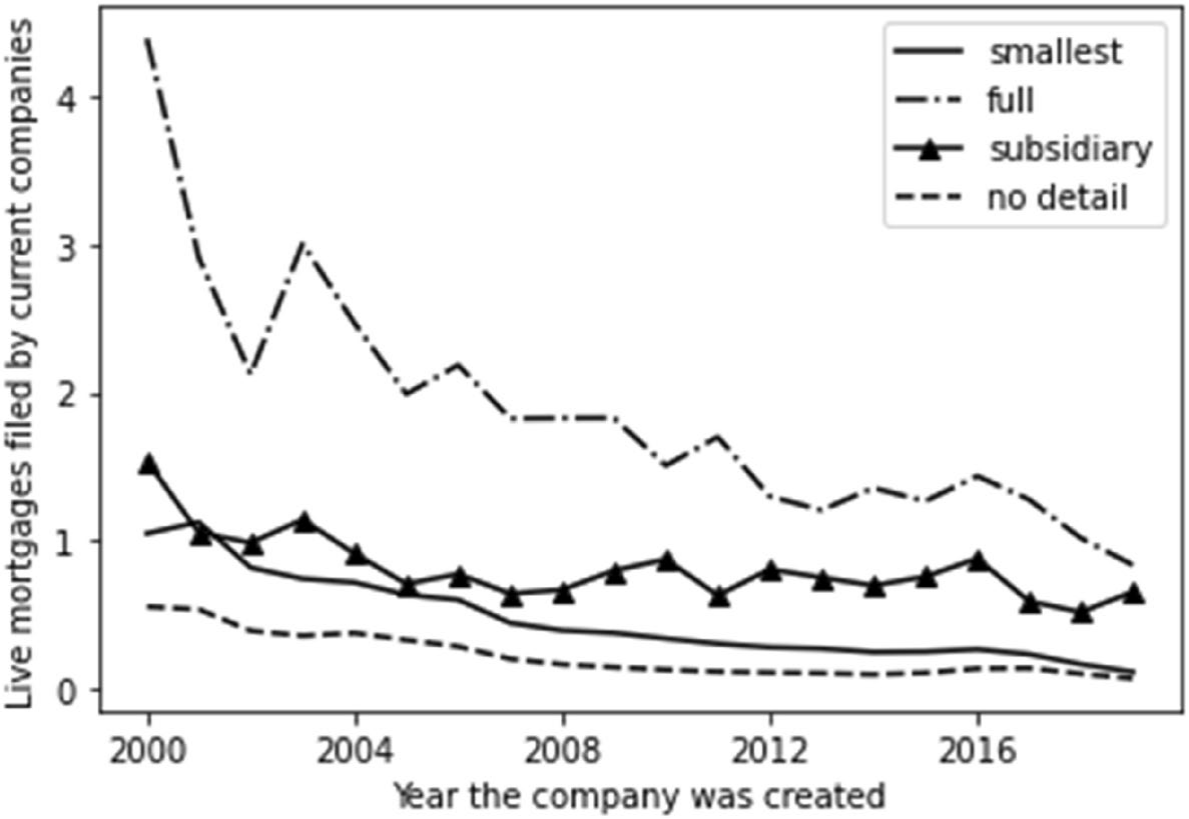
Live mortgages by account type

The same trends are borne out in respect of live mortgages, as set out in Fig. 7 (live mortgages by company type) and Fig. 8 (live mortgages by account type).

Thus, we see two key trends: older companies have granted more mortgages per company on average, and those larger companies under our two proxies have granted more mortgages per company on average. Thus, we have mixed empirical evidence for the need to think small or big first for the corporate finance element of our study: whilst there are more mortgages granted by smaller companies, the larger ones each grant more mortgages over the total of their lives and have more live mortgages per company on average. This provides an important qualification to the otherwise universal need to think small first—we should think big first when it comes to how likely any individual company will be to grant a mortgage.

### Conclusions

Generally, our results indicate a prima facie case for the need to think small first generally in the UK. We see that the vast majority of UK companies are private, and the vast majority either have no accounting information available or fall within the smallest account type. Factoring in time, we see that each of these trends are most acute in the newest companies, which dominate the balance of current companies.

Mortgages and other security rights provide a different picture. Most have been granted by smaller companies, because most companies are smaller. However, larger companies across both metrics are much more likely to each grant more security rights per company than smaller companies are.

## Discussion

### Academics Should Think Small First?

Non-listed companies have long been called the ‘orphan’ of corporate law.^152^ For long it has been argued that the law of unlisted companies requires its own conceptualisation.^153^ This research verifies that not only do we need to pay considerable attention to non-listed companies, which vastly outweigh their listed colleagues by number, we also need to analyse those that qualify to file smaller accounts. It verifies the need to think small first in the UK. It means that concepts such as the market for corporate control^154^ and the separation of ownership and control in listed companies^155^ only apply to a minority of cases. The vast majority of corporate lives by number are private companies. Thus, when academics hold that, for example, ‘the most important issue in corporate governance today’ is that of regulation of dual class shares,^156^ we must ask why that is. It is an issue that will be irrelevant for unlisted companies, which have no current limitations on their ability to issue different share classes.^157^

It is of course possible that bigger companies are more important than smaller companies. However, for academia to proceed on that basis we need two elements that are currently lacking: a clear delineation and a reason why they are more important. Thus, the first page of ‘The Anatomy of Corporate Law’ compares corporate law based upon five characteristics, but states:

[T]he remarkable fact – and the fact that we wish to stress – is that, in market economies, almost all large-scale business firms adopt a legal form that possesses all five of the basic characteristics of the business corporation. Indeed, most small jointly owned firms adopt this corporate form as well, although sometimes with deviations from one or more of the five basic characteristics to fit their special needs.’^158^

It is unclear what constitutes a ‘large-scale business firm’ for corporate lawyers (i.e., how it is delineated from a ‘small-scale business firm’), and why corporate law academics should focus on the larger at the expense of the smaller. There are, of course, a number of metrics that can be easily borrowed from economics and business academia which are available to answer the first. For Chandler, scale related to complexity—a multidivisional firm with a managerial hierarchy was the metric for larger scale.^159^ For Wardley, market capitalisation is the most appropriate metric for scale^160^—although as this only applies to publicly listed companies, its utility for comparing private companies is minimal. Other metrics exist, such as number of employees, or growth, which could be utilised.^161^ The question for corporate law analysis, though, is which metric we consider to be most appropriate and why. This is especially the case as economists and business academics tend to aggregate the discussion of the business together, rather than focus on the entity-by-entity approach usually taken in corporate law: whilst we may have a large business, not every company within that corporate group will necessarily have ‘large’ characteristics. Until we have decided on the appropriate metric, we cannot definitively ascertain which companies qualify as larger companies and which as smaller companies, and so we cannot definitively ascertain the characteristics that apply to such companies.

Based on the heuristic proxies we deployed, though, our research provides prima facie evidence that the UK corporate landscape is focused at the small end of the spectrum. Certainly, if we have 4,608,200 private companies on the public register, and 6,140 public companies, of which roughly 489 are non-investment companies listed on the Main Market of the London Stock Exchange, it seems that we should focus on the largest of the three numbers. This would mean treating matters arising in the smallest companies as the most important for corporate law analysis. It would have to acknowledge that a separation of ownership and control^162^ driving high director/shareholder agency costs,^163^ and considerations as to the requirements of capital markets,^164^ such as prohibitions for dual class shares,^165^ are outliers to the activities of most UK companies. Only a minority of UK corporate vehicles must ever ‘comply or explain’,^166^ never mind worry about the independence of any of their directors.^167^ The traditional mechanics of the market for corporate control^168^ will discipline directors of very few UK companies.

Our research does not mean that there is no value in studying public companies—it may be that on aggregate the few listed companies/companies with large accounts provide more to society (financially, by way of employment/societal impact, etc.) than the aggregate of the smaller companies. For example, it could be that our discipline cares about larger companies because of the power that they exert within the wider economy.^169^ However, it is submitted that this rationale for corporate law analysis focusing on publicly listed companies over private companies needs to be fully justified. Until it is (and any delineation between large and small companies used to make such a case is clarified), we should be loath to ignore the private company, and should perhaps focus our attentions on it—especially when the UK government’s defining mantra for the most recent corporate law reform in the UK was to ‘think small first’.

More, though, our research represents a methodological development in identifying the priorities of company law research. There are two analogies to the development of economic thought that are helpful for company law to reflect on. First, economic thought moved from a qualitative analytical foundation to a quantitative empirical foundation.^170^ The increasing ease in which corporate data can be accessed online and synthesised into large datasets to provide analysis will challenge the traditional approach to company law analysis. Corporate law analysis tends to start from qualitative analysis (mostly economic) and extrapolate to establish what the legal foundation should be,^171^ or start with legal analysis to establish how law appears to influence economic activity (using standard qualitative analytical economic tools).^172^ The ability, though, to use empirical techniques to obtain a quantitative understanding of the behaviour of companies provides another approach to understanding the role of law in regulating the corporate form, arguably one that is more grounded in real-life economic activity than purely analytical qualitative analysis.

For listed companies, this can include testing the adequacy of disclosure to the public market,^173^ reviewing filings that are made under listing rules requiring prompt filings,^174^ or establishing empirically how shareholders vote on corporate matters.^175^ For private companies, this can include seeing the extent to which constitutions deviate from the default form.^176^ In each case, though, evaluation of the law—to judge either the success of new regulation or lacunae in the old regulation—is undertaken by way of empirical analysis. This approach identifies what is actually happening and looks to see whether legal regulation is acting as it should in respect of such activity. It thus proceeds in the opposite manner from traditional corporation law and economics. Given that we have now had 40 years of the latter, it seems that there is more fruitful ground for originality in legal analysis^177^ in extrapolating from empirical data compared to developing an analytical framework. Here, we will see some challenges, though, in the analysis of private companies, as public companies should^178^ and do^179^ have to disclose more information publicly than private companies. This means that such a call to arms risks further skewing to public companies because more information is available about public companies. Accordingly, any empirical analysis will need to reflect on how holistic it can be, and the elements that the boundaries of the studies inherently miss. For example, reviewing disclosures made to the public due to obligations under the listing rules is inherently limited to those companies that are listed. Similarly, whilst our study included all companies in the UK’s corporate database, it was limited to only applying to certain variables. This in no way diminishes the value of the study, merely its extrapolatability to the full UK corporate landscape.

The second analogy to economics further evidences the risk of larger company focus. One of the most enduring legacies^180^ of economist John Kenneth Galbraith is the idea of ‘conventional wisdom’, especially its risk for social science analysis. Galbraith explains the concept thus:

At the highest levels of social science scholarship, some novelty of formulation or statement is not resisted. On the contrary, considerable store is set by the device of putting an old truth in a new form, and minor heresies are much cherished. And the very vigor of minor debate makes it possible to exclude as irrelevant, and without seeming to be unscientific or parochial, any challenge to the framework itself. Moreover, with time and aided by the debate, the accepted ideas become increasingly elaborate. They have a large literature, even a mystique. The defenders are able to say that the challengers of the conventional wisdom have not mastered their intricacies. Indeed, these ideas can be appreciated only by a stable, orthodox and patient man – in brief, by someone who closely resembles the man of conventional wisdom. The conventional wisdom having been made more or less identical with sound scholarship, its position is virtually impregnable. The skeptic is disqualified by his very tendency to go brashly from the old to the new. Were he a sound scholar, he would remain with the conventional wisdom.^181^

It is easy to see the application of this analysis to modern focuses on large and public companies. When the separation of ownership and control is known as the ‘master problem’ of corporate law research, public company matters like the market for corporate control become considered to be the most important, and the focus of the leading work of comparative company law is large companies, the natural conclusion is that these are the most important aspects for corporate law analysis to explore. It is no wonder that closed companies are seen as the orphans of corporate law when more information is available for public companies, and academia focuses on the public and larger company.

How, then, can such a conventional wisdom be challenged? Galbraith reviewed approaches to economic orthodoxy through the examples of the pre-Adam Smith view that wealth maximisation involved accumulating bullion rather than liberal trade, the original kernels of the welfare state at the turn of the twentieth century, and emphasis on balanced budgets at the start of the Great Depression.^182^ He argued:

The enemy of conventional wisdom is not ideas but the march of events. As I have noted, the conventional wisdom accommodates itself not to the world that it is meant to interpret, but to the audience’s view of the world. Since the latter remains with the comfortable and the familiar, while the world moves on, the conventional wisdom is always in danger of obsolescence. This is not immediately fatal. The fatal blow to the conventional wisdom comes when the conventional ideas fail signally to deal with some contingency to which obsolescence has made them palpably inapplicable. This, sooner or later, must be the fate of ideas which have lost their relation to the world.^183^

This research demonstrates a prima facie case that corporate law academia’s focus on larger and public companies is misguided. It does not provide sufficient evidence to replace this conventional wisdom or even fatally wound it—rationales for such focus exist and are likely to be easily argued. However, the assumption that corporate law academic analysis should focus on large and public companies must be justified. As the vast majority of companies on the UK’s corporate database are newer, smaller and private, it seems likely that such a challenge to this conventional wisdom will only grow in strength as time moves on. This matches insights from finance that the role of private companies is increasingly more important in the corporate landscape.^184^ For analysis of public and larger companies to remain dominant in corporate law analysis, such an approach needs now to be overtly justified. If our academic discipline’s conventional wisdom is that larger-scale companies are more important, and this article presents an apparent case against that conventional wisdom, then such wisdom needs to be either justified or abandoned. The march of events here is not a large number of smaller companies—which were always thought to be high by number—but the empirical evidence as to the extent to which they dwarf larger companies, and appear to be increasingly doing so.

### Information for New Companies

One key outcome of our research is the large number of companies for which accounting information is unknown. Third parties may be contracting with the company from its incorporation^185^ but not know the financial position of the company. It is usually argued that creditors can and do self-protect by adjusting to the risk faced with a contracting party.^186^ This lack of information about the vast majority of smaller companies provides a risk generally to third parties, which they are likely to mitigate by increasing the cost to the company (where possible for them to do so).^187^ This would be unnecessary if more information were available. It therefore seems sensible to oblige companies to file information when they pass certain thresholds. These thresholds would seem best to align to the metrics already present for account types: turnover, employees, and balance sheet size. Thus, directors could be obliged to file short statements with Companies House once certain thresholds have been hit, with any filings subject to the same requirements for timing (and liability for falsehoods) as account filings.^188^

Private company regulation is usually adjusted by the creation of new types of vehicles.^189^ However, our evidence unveils evidence that the current form of the UK private company leads to a real information gap. Given that public companies are associated with larger account types, this problem is only felt acutely with private companies: a lack of information is a problem with private companies. As such, it is not a problem that will be identified by those who think big first. Nevertheless, we lack accounting detail for 2,623,442 companies on the public register—56.85% of the company database. As the corporate form is argued to drive riskier behaviour to the detriment of third parties,^190^ this is a large proportion of companies—which (as noted above) are the most likely to enter into insolvency^191^—for which third-party creditors have no access to financial information. Requiring the prompt filing of notices when certain financial metrics are passed prior to the full suite of first accounts would therefore provide vital information for third-party creditors.

It has been argued that corporate filing obligations (on creation and in the company’s early days) drive incorporation choice.^192^ However, more recent research has argued that this is limited to minimum capital requirements,^193^ but that these have a strong effect.^194^ As our proposal would not impose minimum capital requirements, but instead only public filing of information as to financial and employee metrics upon certain threshold triggers, it should not cause incorporations to be driven from the UK. Indeed, as the effect will be to provide more information to third parties, which will help them understand the risk of transacting and thus reduce the need to have a risk premium, it may even be welcomed.

Of course, each company must file a statement of its capital on incorporation,^195^ and frequently over its life—at the very least as part of its annual confirmation statement.^196^ Minimum capital requirements are frequently criticised within the UK sphere on the grounds that there is a disconnect between legal capital and what the funds raised have been used for.^197^ This applies equally to statements of capital—if all funds received have been squandered, then a statement of capital may be actively misleading by implying a healthier financial position than reality. The UK requires directors of public companies to call a meeting of shareholders if the net assets of the public company fall to half of the called-up share capital.^198^ No equivalent exists for private companies. Disclosure processes are said to have different aims between public and private companies.^199^ However, given the risk to third parties posed by the potential insolvency of a small, new company which has not filed much financial information, it would seem sensible to propose a prompt public filing should this occur for private companies prior to the first accounts being filed.^200^ This is even more so if the foregoing reform is followed and companies are required to file details if certain financial thresholds are passed: third parties should be informed of interim successes and failures of companies, rather than waiting a prolonged period of time to find out the extent of a disconnect between the company’s capital and its financial performance.

When coupled with the 1,876,337 companies (40.66% of the corporate register) falling within the smallest account type grouping, over 97% of legal entities on the public database are represented within the ‘smallest’ or ‘no detail available’ requirements for the provision of financial information. Thus, once again, we see that it is a mistake to believe that most companies have to file ‘full’ accounts.

### UK Corporate Governance Should Think Small First

The need to ‘think small first’ is acutely felt when it comes to corporate governance. Even within listed companies, corporate governance of smaller companies tends to be weaker than corporate governance in larger companies.^201^ There is likely to be an even greater gap between those companies whose shares are publicly listed and those whose shares are not, as the UK does not focus on corporate governance of smaller companies. As noted above, there are a number of legal protections which only effectively apply within private or close companies. However, analysis has generally moved from analysis of corporate law to analysis of corporate governance.^202^ Legal analysis of smaller companies needs to follow suit and move to providing corporate governance protections for smaller companies. We also note above a number of governance provisions that will not apply to the majority of companies, because they are contained in a code which is aimed at—and only required for—a subset of publicly traded companies.^203^ A set of corporate governance principles (known as the ‘Wates Principles’, after the chair of the committee that proposed them) exist, but they are expressly targeted at large private companies rather than reflecting a full ‘think small first’ approach.^204^ On the one hand, the flexibility for smaller companies in having no required corporate governance approach reduces their costs. On the other hand, it means that they lack a model for best practice, thus increasing their information costs of trying to find out how best they should run their company. They are not able to merely apply the UK Corporate Governance Code themselves because, as noted above, it aims to protect against concerns not relevant for small companies—it focuses on keeping managers in check (the paradigm issue in a large listed company but likely to be irrelevant for a small company) rather than concerning the relationship between dominant shareholders and the company (the concern for small companies^205^). Some form of corporate governance code for the smallest of companies—even merely as an exemplar of best practice—would thus help reduce the costs in running a small company.

The question then becomes what such corporate governance guidance for smaller companies would look like. Corporate governance is often argued to be primarily of relevance for listed companies,^206^ with attention in respect of smaller companies arising for private companies that want to become listed companies, and therefore need to show a track record of complying with corporate governance requirements for listed companies.^207^ However, the purpose of corporate governance is to provide a check to the dominant power which exists within the company.^208^ As such, whilst the power structures are likely to differ between listed companies (where directors are likely to be powerful) and private companies (where dominance is likely to come from shareholders, although it may technically arise in a number of conflated capacities^209^), the need to protect against *some* dominant power remains in both. In smaller companies, this means protective steps for the minority (who risk suffering due to the actions of the majority^210^) and third parties (who risk suffering due to dominant parties abusing the corporate form^211^). It has been argued that corporate governance is equally important for the long-term success of the company in smaller companies.^212^ Good corporate governance has been seen to exist in smaller companies where a strong institutional shareholder (with repeated experience in the governance of smaller companies) has a representative on the board.^213^ However, for other companies their own governance system is required: even the biggest private companies require private company-specific governance techniques.^214^ Thus, rather than creating a whole new entity type with its own rules (which is the typical response for private business vehicles^215^), we need to promulgate for minorities and third parties what the best governance practices are to provide such countervailing power.

A number of the same techniques can still be deployed in corporate governance, although the mechanics of their operation will need to be changed. For example, for a smaller company, criteria to determine whether a director qualifies as ‘independent’ will need to be tested more vis-à-vis a dominant shareholder than as regards the directors of the company.^216^ The UK Corporate Governance Code currently states that a director will not normally be able to be considered independent if she has one of a series of interactions with the company itself or a director, or ‘represents a significant shareholder’.^217^ Of course, there are many features that could compromise independence which fall short of actively ‘representing’ the dominant party, evidencing that different criteria would be needed for smaller companies to successfully utilise the corporate governance tool of the independent director. Similarly, disclosure requirements can be slightly repurposed to apply outside the listed market.^218^ These are merely illustrations of the wider issue—even where the same corporate governance tools are appropriate for smaller companies, the ways in which those tools need to be deployed will change.

Given that other parties (primarily minority shareholders and third-party creditors) can still suffer from the actions of a dominant shareholder within a smaller company, though, there is an argument for any corporate governance code which ‘thinks small first’ to go beyond mere promulgation of best practice and, instead, have some form of mandatory application to smaller companies despite the additional costs that would arise. In part, these techniques will exist to ensure an adequate separation between the company in question and the dominant party.^219^ Indeed, the key to protecting non-dominant parties in smaller companies is to ensure adequate independence of decision making from the dominant party or parties.^220^ The challenge arises, though, to identify who the dominant party or parties is/are in the operation of the company. Smaller companies now having to disclose^221^ who exerts control over the company can, in theory, help minority shareholders and third parties be aware of who is capable of exerting influence over the company. We now need to provide guidance to them to let them know how control through various constituencies (shareholder, director, etc.) can be mitigated. This will let minority shareholders and third parties establish whether governance procedures are in place which are robust enough to mitigate this risk. As noted above, it may be sufficient to explain how corporate governance tools deployed elsewhere should be repurposed to provide a similar protective function when faced with dominance in a non-listed company.

Two options thus present themselves in respect of the form of such guidance for governance procedures. The existing UK Corporate Governance Code could be adapted to provide considerations for small, private companies as well as listed companies. Then it would more truly reflect its name as it would be applicable to all companies, rather than a small subset of such companies. This approach is taken in South Africa.^222^ There is a risk with this approach, though, that such code ends up sacrificing a tailored approach for one subset of companies to provide a general approach which caters for all companies but in a much vaguer (and therefore less helpful) manner. Alternatively, the UK government could promulgate a separate corporate governance guide for smaller companies, or a number of options to cater for a number of different possibilities as to how smaller companies with different types of dominance could provide adequate protections against that dominance—providing a menu of options to educate where protection may be necessary and how it may look.^223^ Only then could we be stated to think small first in corporate governance terms.

Whichever would be followed, though, the mechanics of enforcement would need to be adjusted if a corporate governance regime for smaller companies were to extend beyond a best practice exemplar. The dominant ‘comply or explain’ model for corporate governance^224^ is predicated upon the concept that the company’s share price will respond to sub-optimal corporate governance.^225^ Whether this mechanism works as intended is debatable,^226^ and even then, it has been argued that some governmental body may be required to objectively judge the quality of disclosures, and discipline recalcitrant companies.^227^ For non-traded companies, there is no such market to perform any such disciplinary function.^228^ As such, a government body would be even more required to judge the quality of disclosures if it was intended that any form of corporate governance code(s) should be considered in any way mandated for smaller companies to encourage corporate compliance. Even then, there is a risk that non-compliance could be tolerated (e.g., a fine paid to maintain poor governance). Here, minority shareholders who had lost out due to a breach of the (relevant) corporate governance code would risk having no remedy. The UK has a remedy available for such minority shareholders—they can petition the court if they suffer unfair prejudice in the running of the company.^229^ UK courts have previously found that predecessors of this regime could be triggered in private companies where inapplicable but analogous public company rules were breached.^230^ It is thus hopeful that any breach of a mandated corporate governance regime would trigger this regime, giving a remedy to minority shareholders. If not, statutory amendments would be required to ensure that any newly applicable corporate governance regime dovetails with this regime to provide a remedy to a minority shareholder.

Overall, then, details remain to be completed, but if we are to truly ‘think small first’ we must do so for corporate governance. This will involve re-examination of the tools used in corporate governance, the precise detail of the deployment of those tools, and enforcement. None of which are currently on the radar for the smallest of companies.

### UK Company Law Legislation Should Think Small First in Form as Well as Substance

We see that small companies are important in the UK landscape, and prima facie evidence of the need to think small first across company law as well as corporate governance. In substance, this is achieved in the accounting context by exempting companies with smaller account types from certain compliance burdens. However, in form, requirements are often worded as an absolute requirement which smaller companies are exempted from. As such, despite the substance of the UK’s reporting requirements meeting a need to ‘think small first’ in the implementation of accounting regulations, the scheme of the UK regulation achieves the opposite in terms of its form. In other words, we have complicated regimes which bigger companies have to apply, with exemptions provided for smaller companies. Whilst we achieve the end of simplifying the process for smaller companies, they have to wade through swathes of requirements that do not apply to them to know that they are exempted.

The UK statutory accounts scheme starts by identifying that different regulations apply to different types of companies,^231^ then stating how a company qualifies as a small or micro entity.^232^ Group accounts must be prepared when faced with a corporate group, although there is a carve-out for small companies.^233^ Similarly, there are requirements to narrate off-balance sheet arrangements, which do not apply to small companies.^234^ Companies must disclose the average number of employees in their accounts, unless they fall within the small company regime.^235^ Company directors must prepare a strategic report into the risks the business faces, unless the company falls within the small company regime,^236^ and also prepare a directors’ report—once more, unless the company falls within the small company regime.^237^ Consistently we see the scheme failing to think small first *in form*. This is highlighted by the emphasis that certain companies are exempt from certain requirements if they fall within certain metrics. This places the burden on smaller companies to understand additional requirements and then ascertain whether they are exempt from them or not. It is thus difficult to tell whether the large number of companies that apply higher requirements to their accounts than required do so on a fully voluntary basis, or merely comply with higher requirements for ease. As 23.75% of companies fall within this category, this appears to be material.

To think small first in form, we would change the ‘base unit’ of corporate type in provisions as to accounts. Rather than setting out what companies must do, and provide a carve-out for smaller companies, we should set out what a smaller company must do. There should then be additional requirements only if certain clear thresholds are met. The substantive outcome would be identical. It would, however, simplify matters for smaller companies, as they would know where they were able to ‘stop reading’ in the regime. Currently, smaller companies must look at all the requirements, and work out which of them they do not have to comply with. A clearer scheme which sets out what micro-companies must provide, then when a company will cease to be considered a micro-company and instead become a smaller company, and then what it must provide, then when a company will cease to be considered a smaller company and instead be considered a medium company, etc., will help ease the process for the vast majority of companies. The UK regime thinks small first in substance, but only by remembering to exclude small companies from regimes. A shift in emphasis to state that certain requirements only apply if a company’s performance exceeds certain metrics, rather than they do not apply if a company’s performance is less than those metrics, will have a real effect for the ease of small company compliance with the regime.

This article makes a clear empirical case for changing the form of reporting requirements accordingly. Often, the economic case is made for corporate law to be a default set of rules: there will be some for which the costs of amending the contract outweigh the benefit (mostly smaller companies), and they will use the default rules; there will be some for which the benefits in a bespoke contract outweigh the costs (mostly larger companies), and they will vary the default rules.^238^ It is often argued that default rules should be set as those which most participants would choose if there were no default rules^239^ as this saves transaction costs.^240^ This must echo for the form of mandatory rules: starting with the form that will apply to most companies, and providing carve-outs or extra requirements for those which are less likely to appear will save transaction costs across the whole system. Given the dominance of smaller companies within the UK landscape, it therefore makes sense that the ‘base unit’ of company should be the smaller company, with additional requirements for larger companies (rather than providing requirements with carve-outs for smaller companies). Companies could, of course, continue to adopt additional reporting should external finance require.^241^ This also reflects the fiscal reality of different sized companies: larger companies will have more resources to understand more complicated and, where necessary, bespoke arrangements. As such, it seems sensible that the costs of understanding what needs to be filtered in or out sits with larger companies rather than smaller companies. The UK’s corporate law scheme therefore needs to be updated to think small first in terms of form as well as substance.

### Rights in Security

We noted above a debate as to whether the grant of a right in security signals financial success or weakness of a company. Our research does not clarify what the *perception* of the grant of security is for third parties. However, it does provide insights into our proxies for size. Whilst most mortgages were granted by smaller companies, this is unsurprising given that smaller companies swamp larger companies by number. On a company-by-company basis, though, a large company is more likely to have granted more mortgages over its life and have more live mortgages outstanding at the date of our study. This could reflect that the larger company has more assets to grant security over: without major corporate assets a creditor may obtain security for repayment by a personal guarantee from directors rather than directly from the asset-light company.^242^ The empirical results outlined here could therefore be merely tautologous: smaller companies have less to grant security over, so will naturally have granted fewer mortgages. The tautology is not perfect, though, as there is not a perfect link between mortgages and assets: floating charges can be granted by companies to secure *future* assets that the company may obtain.^243^ As such, any such personal security is likely to complement the grant of a security interest by the company rather than fully replace it. Accordingly, it is not automatic that mortgages granted will correlate to assets (and, therefore, company size). It could equally reflect a difficulty in smaller companies obtaining structural secured finance. Further research is required to identify why larger companies grant and have granted more mortgages per company than smaller ones. However, there are three immediate implications of the fact that they have.

First, on a company-by-company basis, corporate finance provides an outlier to our general need to think small first. Thus, corporate finance needs to cater for a larger company more than it does for a smaller company. Many mortgages will be granted by smaller companies, but a large company will grant more mortgages than a smaller one. This may provide an insight into a counterargument to the prima facie case for the need to think small first made in this article: that whilst there are more small companies, large companies are more likely to *do* any particular thing. There are two issues with such a claim, though: (a) the argument that any larger company is more likely to do any particular thing relevant to corporate law than a smaller company requires to be advanced and persuasively argued rather than implicitly assumed; (b) the argument is ultimately that the actions of the few more active companies exceed the actions of the many less active companies. More empirical work is needed to establish whether that is true—it should be reiterated that, on aggregate, more mortgages were granted by smaller companies than by larger companies. Second, the risk that secured credit in smaller companies is being abused by majority shareholders^244^ seems low. We did not structurally find that smaller companies granted numerous mortgages, as would be expected if small companies were ritually being abused to obtain external finance and secure the dominant shareholder. Third, each larger company uses rights in security more than each smaller company. Thus, it seems unlikely that the grant of security is inevitably a signal of financial difficulty. Indeed, whilst our research does not state definitively *why* rights in security were granted, nor their signalling effect, the higher number granted per larger company implies positive signalling of the grant of real right in security. Given the dramatic difference between number of mortgages granted per larger company—on both proxies for size—it seems to follow that not only is there a correlation between larger companies and granting more security, but also that this is likely to be appreciated within the marketplace, or at least does not significantly detract from the other perceptions of advantage for interacting with a company.

## Conclusion

Our research evidences an empirical prima facie case to think small first in the UK. The number of private companies dwarfs the number of public companies (which in turn dwarfs the number of those public companies whose shares are listed in London, which in turn dwarfs the number of non-investment companies whose shares are listed on the Main Market). We have no financial information in respect of over half of UK companies, but those for which we do are overwhelmingly within the category of those obliged to file accounts which fall within the smallest category. There is thus an ostensible empirical need to think small first. The exception to this is our corporate finance metric: bigger companies grant more mortgages per company than smaller companies. This, in turn, may provide an important counterargument to our empirical need to think small first: if bigger companies are more likely to do something than smaller companies, then perhaps bigger companies should be studied more than smaller ones. This needs to be persuasively argued rather than assumed. Also needing exploration is whether the aggregate of the activities of fewer bigger companies exceeds the aggregate of the activities of the greater number of smaller companies. This may well be the case, but more research is needed, and until it is undertaken we should be loath to assume that it is.

These trends are exacerbated for newer companies, which are the vast majority of companies on the UK public register. Law makers and academics need to focus their attention more on the plethora of smaller private companies registered within the UK—both the pressures they face and the risk they pose to third parties.

It seems that a large risk is a blind spot as to financial information. Many companies are newer, and there is a time lag before they will have to file financial information, which can be manipulated. Requirements to promptly file financial information outlining when a company exceeds certain metrics, and if their net assets fall below a proportion of their called-up share capital, seem easy wins to begin the mitigation of this blind spot. However, this blind spot arose because insufficient attention was being paid to the dominant calibrations of UK companies. Even if a considerable amount of attention is still paid to publicly listed companies in the UK, much more needs to be paid to the smaller ones. This will help identify and resolve issues such as that outlined in this research—where we have no complete financial information available for over half the business vehicles on the UK’s corporate database. We provide no corporate governance requirements (or even guidance) for smaller companies in the UK, exposing two related gaps in respect of the promulgation of best practice, and also remedies for those harmed by failures to comply with such best practices.

The outcomes of this study may, perhaps, be unsurprising, and verify hunches and tacit assumptions as to the operation of the corporate landscape. This verification, though, is vitally important to prove that which has been tacitly assumed. More than this, it foregrounds such assumptions and their implications for corporate law scholarship. This asks wider questions of corporate law as a discipline. We have explored a dataset that the UK corporate registry releases every month. It is not the only dataset that is published by this registry. Similarly, the database is publicly searchable. This creates a wealth of possibilities for the empirical analysis of UK company law. It remains to be seen whether corporate law follows economics in moving from qualitative analytical study to quantitative empirical study, but the potential is there should the discipline wish. Given that so much of corporate law analysis is predicated upon economic analysis, it seems incongruous to refuse to take this step.

## Data Availability

The dataset is based on data publicly available from the UK public registry Companies House. Readers are able to access this registry for free. The methodology applied to the public information to produce the information is outlined in this article, and the article contains a link to a workbook outlining in further detail the coding that we employed. Our methodology can be accessed at https://github.com/guillemram97/companiesdata to enable all readers to access it.

## Footnotes

1 Companies Act 2006.

2 Department for Trade and Industry (2005), ch. 4; Arden (2002).

3 Lowry (2009), p 609.

4 Department for Trade and Industry (2005), para. 4.1. See also Harris (2013).

5 Department for Trade and Industry (2005), para. 4.1. See also Freedman (2003).

6 Department for Trade and Industry (2005), para. 4.2. Compare Companies Act 1985, Part XI, ch. IV, with Companies Act 2006, Part 13. See also Boros (2003).

7 Department for Trade and Industry (2005), para. 4.2. See Hardman (2021a).

8 Department for Trade and Industry (2005), para. 4.8. See Xiong (2020).

9 Department for Trade and Industry (2005), para. 4.10.

10 Ibid., para. 4.9. For this operation in practice, see discussion in Hardman (2021b).

11 European Commission (2008).

12 Beaupérin (2014).

13 Pereira (2021).

14 In Sect. 4.1 we explore the academic focus on larger companies, and argue that whilst it may be highly justifiable, such focus needs to be properly delineated and justified. In Sect. 4.4, we explore the UK legislative schema in respect of corporate accounts, and argue that whilst it succeeds in thinking small first in substance (smaller companies have to do less than larger companies), it fails to do so in form, as it tends to start with full requirements and then provide carve-outs.

15 Dignam and Oh (2019), p 19.

16 https://www.gov.uk/government/statistics/incorporated-companies-in-the-uk-april-to-june-2021n (accessed 25 November 2021).

17 http://download.companieshouse.gov.uk/en_output.html (accessed 25 November 2021).

18 Companies Act 2006, s. 4.

19 Ibid., Part 5.

20 Ibid., s. 1066. See discussion in Hardman (2021a).

21 Companies Act 2006, s. 86.

22 Ibid., Part 15.

23 Ibid., Part 25. See discussion in MacPherson (2019).

24 The dataset in question ran to nearly 5,000,000 rows. This created a number of operational challenges—such as the need for adequate hardware, a longer time for processing, difficulties in choosing and designing the best visualisation of the data, etc.

25 Dignam and Oh (2019).

26 LoPucki (2018).

27 We discuss this in further detail in Sect. 4.1.

28 http://download.companieshouse.gov.uk/en_output.html (accessed 25 November 2021).

29 Companies Act 2006, s. 7.

30 Ibid., s. 1000.

31 Ibid., s. 1003.

32 Insolvency Act 1986, s. 201.

33 See Berry (2021); Hardman (2021c).

34 See https://github.com/guillemram97/companiesdata (accessed 18 May 2022).

35 Companies Act 2006, s. 4(2). See Hardman (2021a).

36 Companies Act 2006, s. 3.

37 Ibid., s. 3.

38 Ibid., s. 3.

39 Ibid., s. 60.

40 Ibid., s. 58.

41 Ibid., s. 755.

42 Freedman (1994) talks of a spectrum, in which unlisted public companies sit between a large private company and a company whose shares are publicly listed, p 559.

43 Companies Act 2006, s. 755(1)(b); UK Listing Rules, Rule 2.2.1R, https://www.handbook.fca.org.uk/handbook/LR.pdf (accessed 25 November 2021).

44 Companies Act 2006, s. 6. See Liptrap (2021).

45 Limited Liability Partnerships Act 2000. See Finch and Freedman (2002); Freedman and Finch (1997).

46 Limited Partnership Act 1907. See Hardman (2021c); Berry (2021); Berry (2019).

47 Legislative Reform (Private Fund Limited Partnerships) Order 2017 (SI 2017/514). See Morris (2017).

48 See Davies et al. (2021), paras. 1-006–1-012.

49 See, for example, Picton (2021); Dunn (2014); Sørensen and Neville (2014); Lombardo (2013); Fici (2016).

50 E.g., Squire (2021); Wells (2021); Ribstein (2009).

51 Companies Act 2006, ss. 90–96.

52 Ibid., s. 97–s. 101. This is commonly undertaken in the UK after the shares in the company have been acquired by one entity, such as in private equity transactions.

53 Companies Act 2006, ss. 102–111.

54 Companies Act 2006, Part 7.

55 For example, see Cosh and Hughes (1994), p 18.

56 See Hardman (2021a).

57 For example, see Ferran (2019), pp 527-528.

58 Berry (2019).

59 Companies Act 2006, s. 761. See Armour (2006).

60 Ibid., s. 336.

61 Ibid., s. 200.

62 Kraakman et al. (2017), p 10.

63 Under UK Corporate Governance Code 2018, https://www.frc.org.uk/directors/corporate-governance-and-stewardship/uk-corporate-governance-code (accessed 25 November 2021).This is not an absolute requirement, but instead operates on a ‘comply or explain’ basis—see discussion in Keay (2014).

64 E.g., Manne (1965).

65 E.g., Fama (1980).

66 Thompson (1990).

67 E.g., Grundfest (1992); Easterbrook and Fischel (1985).

68 Romano (1984).

69 Berle (1962).

70 Berle and Means (1967).

71 Companies Act 2006, s. 755.

72 Wells (2008), p 273.

73 Hardman (2022e).

74 Fama and Jensen (1985).

75 E.g., Easterbrook and Fischel (1986); Hetherington and Dooley (1977); Milman (2017).

76 Eisenberg (1989), p 1463.

77 Prentice (1983); Fleischer (2018).

78 Cheffins (1989).

79 Schwartz and Scott (2003).

80 E.g., Claessens and Tzioumis (2006).

81 This has been analogised most cogently in the context of close companies—see Fama and Jensen (1983); Kulms (2001).

82 E.g., Mahoney (2000).

83 E.g., Eisenberg (1989), pp 1463-166.

84 E.g., veil piercing – Thompson (1991), and court-based minority protections—Payne (2005), pp 674-675.

85 Milman (2017).

86 Fleischer (2018); Moore and Petrin (2017), pp 10-16.

87 See discussion in Gözlügöl and Ringe (2022), and further discussion in Sect. 4.3 below.

88 See Bryer (1993).

89 See Hardman (2022a).

90 Companies Act 2006, s. 391. See discussion in Wan (2008).

91 Companies Act 2006, s. 392.

92 Ibid., s. 442. This was temporarily delayed due to the coronavirus pandemic—see Corporate Insolvency and Governance Act 2020, s. 37, Sch. 14.

93 Companies Act 2006, s. 396. Walker-Arnott (2017).

94 See Companies Act 2006, s. 830, as discussed in Ferran (2019); Armour (2006).

95 E.g., Corporation Tax Act 2009, s. 2. The filing processes are separate, and there are slightly different requirements for the tax calculation compared to the public filing obligation, but UK authorities are simplifying filing accounts for both purposes with one submission—see https://www.gov.uk/prepare-file-annual-accounts-for-limited-company/file-your-accounts-and-company-tax-return (accessed 25 November 2021).

96 E.g., Ormrod and Cleaver (1993).

97 Flores (2011). Although whether this achieves the desired ends is frequently debated—e.g., Arruñada (2004); Eisenberg and Macey (2004).

98 See carve-outs in Companies Act 2006, ss. 381–384B, 394A–394C, and 465–467.

99 Ibid., s. 465.

100 Ibid., s. 382, s. 384.

101 Ibid., s. 384A.

102 Ibid., s. 384B.

103 Full and medium companies are governed by the Large and Medium-sized Companies and Groups (Accounts and Reports) Regulations 2008 (SI 2008/410) (as amended), small companies are governed by the Small Companies and Groups (Accounts and Directors’ Reports) Regulations 2008 (SI 2008/409) (as amended). Micro companies are governed by the Small Companies (Micro-Entities’ Accounts) Regulations 2013 (SI 2013/3008).

104 Companies Act 2006, s. 474(1). Group structures are pervasive in order to ensure that liabilities and assets are placed to the best advantage of the overall structure—see Squire (2011).

105 Companies Act 2006, s. 399. Consolidation involves looking at the corporate group as a whole in addition to the financial performance of each component part of that group—see Wooldridge (1988).

106 Companies Act 2006, s. 1169.

107 Ibid., s. 394A and s. 448A.

108 Ibid., s. 394C and s. 448C.

109 Small Companies and Groups (Accounts and Directors’ Report) Regulations 2008, Sch. 1, para. 1A.

110 Companies Act 2006, s. 382.

111 Ibid., s. 836. See discussion of the general position in Rickford (2006); Tham (2005).

112 Companies Act 2006, s. 393. See discussion in McGee (1991); Zahid (2008).

113 House of Commons (2018), p 3.

114 See Hensher et al. (2007); Chen and Lee (1993); Lensberg et al. (2006).

115 Companies Act 2006, s. 390.

116 Ibid., s. 442.

117 Ibid., s. 392.

118 Ibid., s. 442.

119 Ibid., s. 451.

120 The application of the fine is automatic, and ratchets quite dramatically—see https://www.gov.uk/annual-accounts/penalties-for-late-filing (accessed 25 November 2021).

121 Technically, the registrar can strike off a company which it believes is not carrying on business—Companies Act 2006, s. 1000. In the case of late filing of accounts, the registrar interprets this as having two reminder letters ignored—see https://www.gov.uk/government/news/companies-house-to-resume-the-compulsory-strike-off-process (accessed 25 November 2021).

122 LoPucki (2018).

123 Companies Act 2006, s. 16. See Watson (2019).

124 Dodd (1932), p 1160.

125 E.g., Evans (1987); Coad et al. (2013).

126 Companies Act 2006, s. 1000 and s. 1003; Insolvency Act 1986, s. 201.

127 See Goode and Gullifer (2017), para. 1–54; Waddilove (2014).

128 See discussion in Davies (2013); Sheehan (2006); Armour (2004).

129 Companies Act 2006, s. 859A; MacPherson (2019).

130 See Gretton (1987) for a more technical discussion of real rights in security.

131 E.g., Jackson and Kronman (1979); White (1984).

132 E.g., LoPucki (1994); Hudson (1995); Schwartz (1984).

133 Levmore (1982).

134 Hardman (2022b).

135 Companies Act 2006, s. 859A(4).

136 Ibid., s. 859F(1).

137 Ibid., s. 859F(2).

138 Ibid., s. 859F(3).

139 Ibid., s. 859H. In addition, failing to register a security makes the obligation secured immediately payable (ibid., s. 859H(4)), incentivising the security granter to register the security.

140 Bebchuk and Fried (1996); Bebchuk and Fried (1997).

141 Security can always be released upon the repayment of the relevant debt under English law – see Berg (2002).

142 Companies Act 2006, s. 859L(3).

143 Ibid., s. 859L(1).

144 See Hardman and MacPherson (2022).

145 As security ranks, in the absence of agreement between secured parties to the contrary, on a ‘prior in time, earlier in right’ approach. See discussion in Jansen (2002).

146 See https://github.com/guillemram97/companiesdata (accessed 18 May 2022).

147 Hardman (2022c).

148 This number should be treated with caution—as noted above, the register for limited partnerships currently contains defunct vehicles.

149 Watson (2019).

150 See discussion in Sørensen (2021).

151 See the base figures available at https://github.com/guillemram97/companiesdata (accessed 18 May 2022). Figures and tables in this section were created by us in accordance with the visualisation techniques set out at this link.

152 Chayes (1960); Wells (2008); Bachmann et al. (2014), p 32.

153 E.g., Manne (1967).

154 Manne (1965).

155 Berle and Means (1967); Romano (1984).

156 Coffee (2018).

157 As, in the UK, unlisted companies are already free to issue dual class shares—a company is merely unable to do so and be listed on the London Stock Exchange’s premium tier—see Reddy (2020).

158 Kraakman et al. (2017), p 1.

159 See Chandler (1977), especially ch. 9, ch. 13 and ch. 14; Chandler (1994), especially ch. 2. For discussion of some aspects of Chandler’s work in modern corporate law, see Johnston et al. (2019).

160 Wardley (1991).

161 See the overview in Barnes et al. (2019).

162 Berle and Means (1967).

163 E.g., Fama (1980).

164 E.g., Grundfest (1992).

165 E.g., Reddy (2020).

166 E.g., Keay (2014).

167 E.g., Clark (2007); Gutiérrez and Sáez (2013); Ringe (2013).

168 Manne (1965).

169 Hannah (1983), especially ch. 11; Parkinson (1995), especially ch. 1, ch. 5 and ch. 11.

170 See discussions in Copeland (1951); Redlich (1957).

171 E.g., Mahoney (1995).

172 E.g., Gilson and Kraakman (1984).

173 E.g., Esser et al. (2018); Esser et al. (2020).

174 E.g., Davies (2019).

175 E.g., Christie (2022).

176 E.g., Hardman (2021a).

177 See discussion in Siems (2008).

178 E.g., Stout (2003); Levmore (2003).

179 See discussion in Rauterberg (2021). For a domestic example, see the UK’s retained version of the Market Abuse Regulation (European Union (Withdrawal) Act 2018), Article 17 requires public disclosure of inside information.

180 See Dunn and Pressman (2006); Reisman (1990).

181 Galbraith (1999), p 9.

182 Ibid., ch. 2.

183 Ibid., p 11.

184 E.g., Stulz (2020); Asker et al. (2015); Doidge et al. (2018).

185 But not before in the UK – Companies Act 2006, s. 51. For the position pre the 2006 Act, see Pennington (2002).

186 E.g., Easterbrook and Fischel (1989).

187 On the adjustment of pricing for risk, see Bebchuk and Fried (1996); Bebchuk and Fried (1997).

188 Of course, any timing requirements would need to be linked to awareness (coupled with an obligation to promptly become aware), rather than when the company actually passed the threshold.

189 Hansmann et al. (2009); McCahery et al. (2009).

190 E.g., Goodhart and Lastra (2020); Hansmann and Kraakman (1991).

191 See Hensher et al. (2007); Chen and Lee (1993); Lensberg et al. (2006).

192 Djankov et al. (2002).

193 van Stel et al. (2007).

194 Braun et al. (2012); Becht et al. (2008).

195 Companies Act 2006, s. 10.

196 Ibid., s. 853D.

197 E.g., Armour (2006).

198 Companies Act 2006, s. 656.

199 E.g., Hardman (2021b).

200 It may be advantageous to require such filing even afterwards to protect third parties, but that goes beyond the scope of our study.

201 The most recent analysis and summary of research is contained in Kastiel and Nili (2022).

202 See overview in Gilson (2018).

203 UK Corporate Governance Code 2018, p 3, https://www.frc.org.uk/directors/corporate-governance-and-stewardship/uk-corporate-governance-code (accessed 13 June 2022). This is a common feature of such codes worldwide—see Fleischer (2018).

204 See Financial Reporting Council (2018), p 3; Kokkinis and Sergakis (2020), para. 3.4.

205 Hardman (2022d).

206 Moore and Petrin (2017), pp 10–16.

207 See Lund and Pollman (2021).

208 Cheffins (2018); Tricker (2011).

209 E.g., manager, shareholder and employee—see Eisenberg (1995).

210 See Hardman (2022d).

211 The potential for the corporate form to be abused to the detriment of third parties is explained in Hansmann and Kraakman (1991); Goodhart and Lastra (2020).

212 McCahery et al. (2013); Witney (2020), ch. 9 and ch. 10.

213 Lerner (1995); Pollman (2020).

214 Fan (2016).

215 McCahery and Vermeulen (2001), p 873.

216 For the need for such a switch, see Gilson (2006).

217 UK Corporate Governance Code 2018, p 3, https://www.frc.org.uk/directors/corporate-governance-and-stewardship/uk-corporate-governance-code,provision10 (accessed 13 June 2022).

218 Gözlügöl and Ringe (2022).

219 To ensure that, operationally, the separation between the dominant party and the corporate fund is respected—Watson (2018).

220 Hardman (2022d).

221 Under the ‘Persons of Significant Control’ regime—Companies Act 2006, Sch. 1A.

222 Esser and Delport (2018).

223 E.g., Klausner (1995), p 839.

224 See MacNeil and Esser (2022).

225 Sergakis (2015).

226 E.g., Moore (2009).

227 Keay (2014).

228 Hetherington and Dooley (1977).

229 Companies Act 2006, s. 994.

230 *Re A Company* [1986] BCLC 382.

231 Companies Act 2006, s 380.

232 Ibid., ss. 381–384B.

233 Ibid., s. 399.

234 Ibid., s. 410A.

235 Ibid., s. 411.

236 Ibid., s. 414B.

237 Ibid., s. 415A.

238 Perhaps the most famous case for which is made in Easterbrook and Fischel (1989).

239 See Johnston (1990); Ayres and Gertner (1992); Hardman (2021a).

240 Posner (2003), p 839.

241 Which appears prevalent for private companies—see Sect. 3.1.2 above, and Hope and Vyas (2017).

242 Armour (2006).

243 See Pennington (1960); Ferran (1988).

244 As argued to have occurred in the seminal (Harris (2013)) case of *Salomon v A Salomon & Co Ltd* [1896] UKHL 1; [1897] AC 22.
